# Design, synthesis, in vitro, and in silico biological evaluations of coumarin-indole hybrids as new anti-α-glucosidase agents

**DOI:** 10.1186/s13065-022-00882-2

**Published:** 2022-11-03

**Authors:** Davood Rezapour Niri, Mohammad Hosein Sayahi, Somayeh Behrouz, Ali Moazzam, Somayeh Mojtabavi, Mohammad Ali Faramarzi, Bagher Larijani, Hossein Rastegar, Maryam Mohammadi-Khanaposhtani, Mohammad Mahdavi

**Affiliations:** 1 Medicinal Chemistry Research Laboratory, Department of Chemistry, Shiraz University of Technology, Shiraz, Iran; 2 Department of Chemistry, Payame Noor University (PNU), Tehran, Iran; 3 Endocrinology and Metabolism Research Center, Endocrinology and Metabolism Clinical Sciences Institute, Tehran University of Medical Sciences, Tehran, Iran; 4 Department of Pharmaceutical Biotechnology, Faculty of Pharmacy, Tehran University of Medical Sciences, Tehran, Iran; 5 Cosmetic Products Research Center, Iranian Food and Drug Administration, MOHE, Tehran, Iran; 6 Cellular and Molecular Biology Research Center, Health Research Institute, Babol University of Medical Sciences, Babol, Iran

**Keywords:** Coumarin, Indole, α-Glucosidase, Design, Synthesis, Hybrid

## Abstract

**Background:**

A series of coumarin-indole hybrids was synthesized as the new α-glucosidase inhibitors. The title hybrids were considered as α-glucosidase inhibitors because had two active pharmacophores against α-glucosidase: coumarin and indole.

**Methods:**

The thirteen various derivatives **4a**–**m** were synthesized, purified, and fully characterized. These compounds were evaluated against α-glucosidase in vitro and in silico. In silico pharmacokinetic studies of the most potent compounds were also performed.

**Results:**

Most of the title compounds exhibited high anti-α-glucosidase activity in comparison to standard drug acarbose. In particular, the phenoxy derivative **4d** namely 3-((1*H*-indol-3-yl)(3-phenoxyphenyl)methyl)-4-hydroxy-2*H*-chromen-2-one showed promising activity. This compound is a competitive inhibitor against α-glucosidase and showed the lowest binding energy at the α-glucosidase active site in comparison to other potent synthesized compounds and acarbose.

**Conclusion:**

Compound **4d** can be a lead compound for further structural development to obtain effective and potent α-glucosidase inhibitors.

**Supplementary Information:**

The online version contains supplementary material available at 10.1186/s13065-022-00882-2.

## Introduction

Type 2 diabetes (T2DM) is a metabolic disorder which is considered as a serious chronic health condition [1]. The prevalence of this disorder is increasing in worldwide due to false lifestyle patterns such as physical inactivity and incorrect nutrition [2]. This disease, if untreated, can lead to serious problems including kidney failure, blindness, cardiovascular diseases, and nerve damage [3]. Considering the limitations of current therapies such as adverse side effects and high secondary failure rates, there are a lot of demands for the design and development of new drugs for treatment of T2DM. Inhibition of carbohydrate degrading enzymes such as α-glucosidase is one of the therapeutic goals for T2DM treatment [4]. α-Glucosidase is an intestinal enzyme that convers carbohydrates to glucose and plays a key role in increasing postprandial blood glucose level [5]. α-Glucosidase inhibitors have been widely prescribed to treat of T2DM. These medications often increased secretion of undigested starch into the colon and thus their use is associated with a variety of undesirable gastrointestinal symptoms. For instance, acarbose as the most widely used drug in this category causes diarrhea, bloating, flatulence, and abdominal discomfort in nearly 20% of patients [6].

**Fig. 1:**
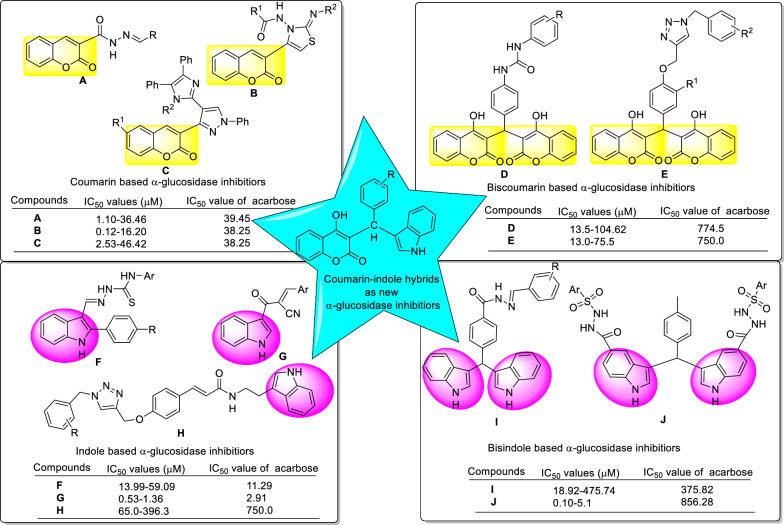
Rationale for the design of coumarin-indole hybrids as the new α-glucosidase inhibitors. Therefore, based on anti-α-glucosidase agents containing coumarin (compounds **A**–**C**), biscoumarin (compounds **D**–**E**), indole (compounds **F**–**H**), or bisindole (compounds **I**–**J**), we considered coumarin-indole hybrids as new α-glucosidase inhibitors. These compounds after synthesis, evaluated against α-glucosidase in vitro and in silico

Coumarin ring has extensive utilization in design of new bioactive compounds [7]. Many natural and synthetic derivatives of coumarin with various remarkable bioactivities such as antibacterial, anticancer, anti-Parkinson, anti-HIV, and anti-proliferative activities have been reported [8–10]. This ring also found in the several series of synthetic potent α-glucosidase inhibitors such as compounds **A**–**C** (Fig. 1) [11–13]. Furthermore, interestingly, derivatives containing two coumarin rings such as biscoumarins **D** and **E** also exhibited high inhibitory activity against α-glucosidase (Fig. 1) [14, 15].

Indole is a bicyclic heterocyclic ring with considerable applications in medicinal chemistry and crucial role in the biological systems [16]. Indole scaffold composed of a benzene ring fused to pyrrole ring. This ring is found in many natural derivatives such as plant alkaloids, fungal metabolites, and marine natural products [17]. Indole is also involved in the formation of amino acids, growth hormones, and alkaloids [18]. There are the several drugs containing indole ring with treatment applications such as anti-cancer, anti-hypertensive, and antimitotic activities in the pharmaceutical market [19, 20]. Recent studies showed that indole ring had attracted much attention for design of effective structures for targeting of α-glucosidase [21]. In this regards, several series of synthetic indole or bisindole based α-glucosidase inhibitors have been reported (Fig. 1, compounds **F**-**J**) [22–26].

## Results and discussion

### Chemistry

**Scheme 1.**
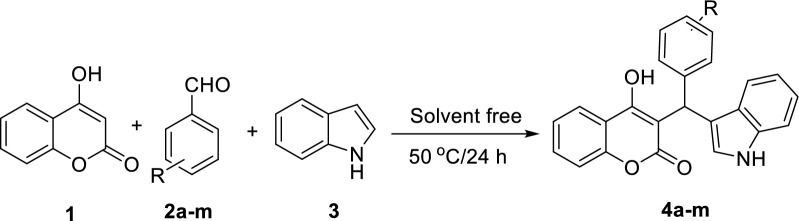
Synthesis of coumarin-indole derivatives **4a**–**m**

The coumarin-indole derivatives **4a–m** were prepared according to Scheme 1 in the excellent yields (79–87%) [27–30]. These compounds were synthesized via a simple one-step reaction of 4-hydroxycoumarin **1**, benzaldehyde derivatives **2a–m**, and 1*H*-indole **3** in the solvent free condition at 50 °C.

### Anti-α-glucosidase activity and SAR discussion

The in vitro anti-α-glucosidase activity of the target compounds **4a–m** was evaluated against yeast form of this enzyme, in comparison with acarbose as a positive control and the obtained IC_50_ values are listed in Table 1.

**Table 1:** Structures and IC_50_ values (µM) of compounds **4a**–**m** against yeast α-glucosidase


Compound	R	IC_50_ (µM)^a^	Compound	R	IC_50_ (µM)^a^
**4a**	H	118.0 ± 3.1	**4h**	4-F	474.9 ± 2.6
**4b**	4-CH_3_	167.5 ± 0.8	**4i**	3-Cl	> 750
**4c**	4-OCH_3_	170.2 ± 2.1	**4j**	4-Cl	229.7 ± 2.3
**4d**	3-Phenoxy	116.0 ± 0.7	**4k**	3-Br	> 750
**4e**	3-OH	> 750	**4l**	2-NO_2_	> 750
**4f**	4-OH	> 750	**4m**	3-NO_2_	180.5 ± 1.4
**4g**	3-F	174.0 ± 2.3	**Acarbose**	–	750.0 ± 5.0

^a^Results were reported as mean ± SD (n = 3)

As listed in Table 1, the general structure of hybrid derivatives of coumarin and indole moieties was varied by substituents on pendant phenyl ring between the latter moieties. As evidenced from IC_50_ values, the most potent compounds were 3-phenoxyphenyl derivative **4d** and un-substituted phenyl derivative **4a**. These compounds were around sixfold more potent than acarbose. Evaluation on other derivatives with electron-donating substituents demonstrated that 4-methyl and 4-methoxy derivatives **4b–c** also exhibited high inhibitory activity against α-glucosidase while introduction of hydroxy substituent on the pendant phenyl ring, as in case of compounds **4e** and **4f**, led to loss of effect. SAR evaluation of derivatives **4g–m** with electron-withdrawing substituents revealed that the best effects obtained with fluoro and nitro substituents in 3-position of the pendant phenyl ring (compounds **4 g** and **4m**, respectively). Movement of fluoro substituent of 3- to 4-position led to a dramatically decrease in inhibitory activity (compound **4h**) while movement of nitro substituent of 3- to 2-position completely abolished anti-α-glucosidase activity (compound **4l**). The third potent compound among the compounds containing electron-withdrawing substituent was 4-chloro derivative **4j**. Changing the position of this substituent to 3-position led to loss of effect as observed in 3-chloro derivative **4i**. Like to 3-chloro derivative, 3-bromo derivative (compound **4k**) also did not show activity against α-glucosidase (Additional file 1).

According to SAR study, in general, it should be mentioned that in addition to the type of substitution, the position of the substitutions has a significant effect on the observed inhibitory activities against α-glucosidase.

### Kinetic study

**Fig. 2:**
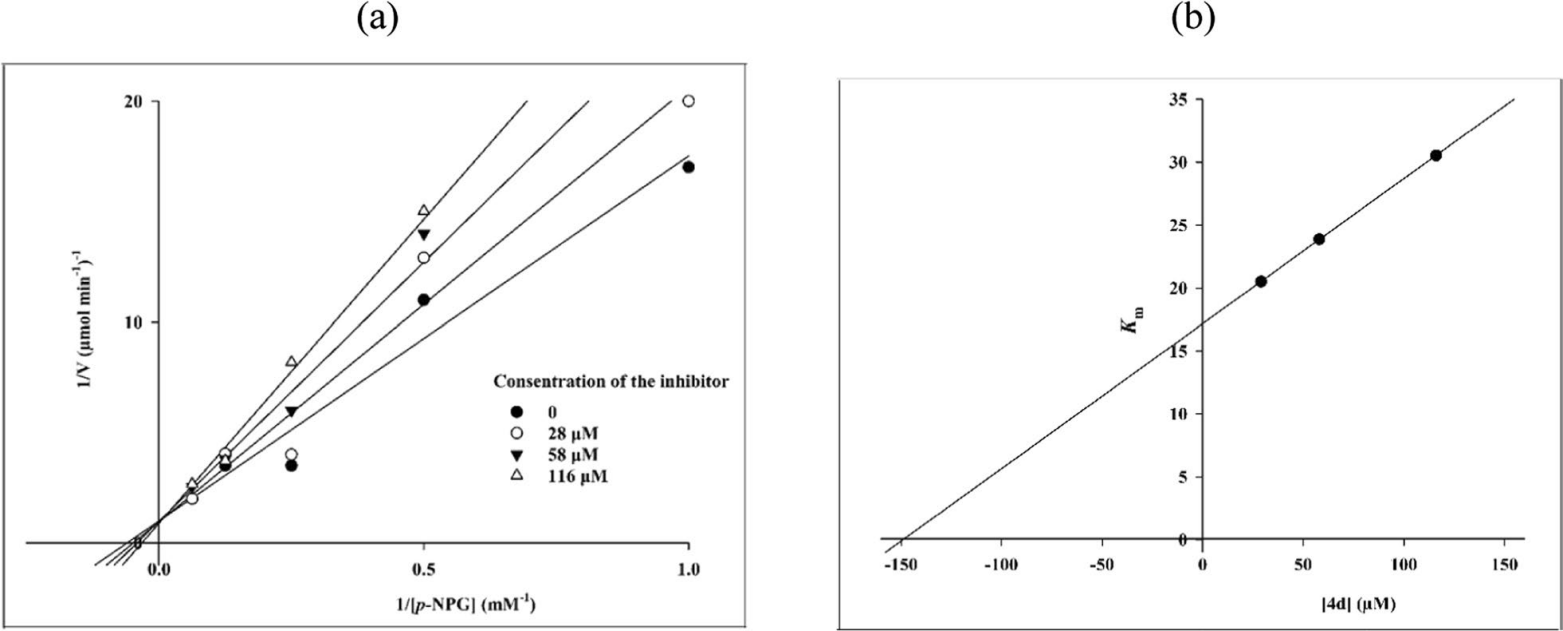
Kinetic study of compound **4d** into α-glucosidase. **a** The Lineweaver–Burk plot in the absence and presence of different concentrations of compound **4d**; **b** The secondary plot between *K*_m_ and various concentrations of compound **4d**

To determine the mechanism of α-glucosidase inhibition of the newly synthesized compounds, the kinetic study was performed on compound **4d** as representative compound. The relative velocity of the α-glucosidase was determined on four increasing concentrations of the *p*-nitrophenyl glucopyranoside as substrate. To construct the Lineweaver–Burk plot, the enzyme velocity was calculated in the presence of compound **4d** as inhibitor at following concentrations: 0, 28, 58 and 116 µM. Then, the Lineweaver-Burke plot was depicted using the reciprocal of velocity and substrate concentration (Fig. 2a). Based on the obtained plot, a competitive type of inhibition by compound **4d** was observed. Using by the Lineweaver–Burk secondary plot (Fig. 2b), a K_i_ value equal to 148 µM was determined for compound **4d**.

### Docking study

**Fig. 3:**
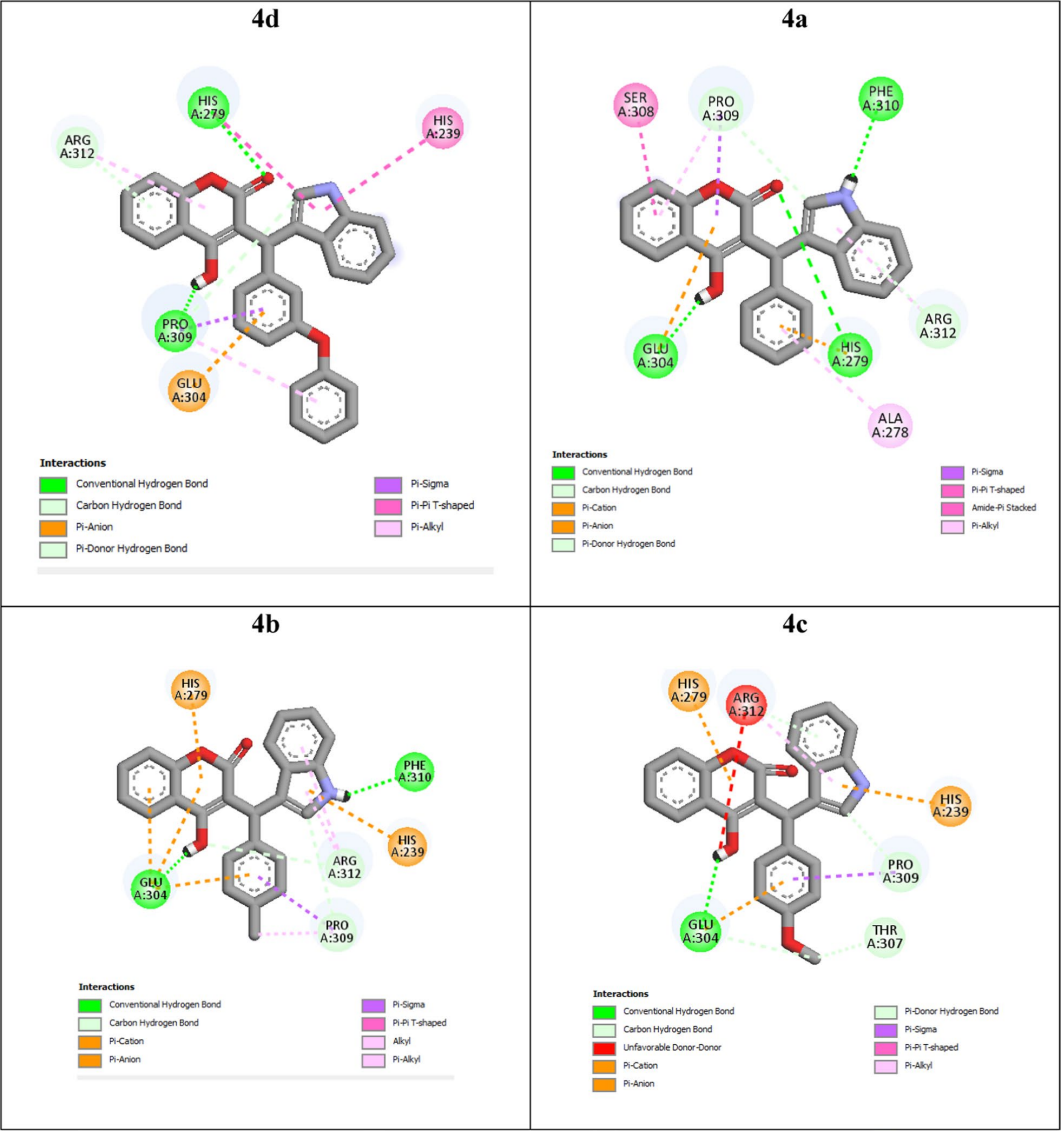
View of the two-dimensional structure of ligand binding cavity of the modeled α-glucosidase with the docked compounds **4d**, **4a**, **4b**, and **4c** visualized in the BIOVIA Discovery Studio v.3.5

The molecular modeling was performed to gain insight into the binding modes of coumarin-indole derivatives to the conceivable target enzyme (α-glucosidase, modeled form) [31]. The most potent compounds **4a–d** were docked at α-glucosidase active site and the best docked poses in terms of the binding energy (BE) were selected. The interaction modes of the latter compounds were shown in Fig. 3. BE values of compounds **4d**, **4a**, **4b**, **4c**, and acarbose in the α-glucosidase active site were − 9.08, − 8.65, − 8.61, − 8.26, and − 4.04 kcal/mol, respectively. These BE values suggested high affinities to the active site in the new α-glucosidase inhibitors **4d**, **4a**, **4b**, and **4c** in comparison to acarbose. The order of BEs in the selected compounds **4d**, **4a**, **4b**, and **4c** is in agreement with the obtained in vitro inhibitory activities of these compounds.

Hydroxy and carbonyl units of coumarin ring in the most potent compound **4d** attached to Pro309 and His279, respectively, through hydrogen bonds (Fig. 3). His279 and His239 formed two π-π stacking interactions with indole ring. Moreover, a π-anion interaction also observed between pendant phenyl ring of compound **4d** and Glu304. Furthermore, several hydrophobic interactions with residues Pro309 and Arg312 and a none-classical hydrogen bond with the latter amino acid were observed in the binding mode of compound **4d**.

The second potent compound **4a** established three classical hydrogen bonds with Glu304 (hydroxy group), His279 (carbonyl unit), and Phe310 (NH unit) and two none-classical hydrogen bonds whit Arg312 and Pro309 (Fig. 3). Compound **4a** also formed a π-anion interaction with Glu304 (coumarin ring) and a π-cation interaction with His279 (pendant phenyl ring). This compound also attached to residues Ser308, Ala279, Pro309, and Arg312 through hydrophobic interactions.

As can be seen in Fig. 3, the third potent compound **4b** formed hydrogen bonds with residues Glu304 (hydroxy group) and Phe310 (NH unit). This compound formed several π-ion interactions with Glu304 (two π-anion interactions with coumarin ring and a π-anion interaction with pendant 4-methyphenyl ring), His239 (a π-cation interaction with indole ring), and His279 (a π-cation interaction with coumarin ring). Furthermore, hydrophobic and none-classical hydrogen bonds between this compound and residues Arg312 and Pro309 are also observed.

The fourth potent compound **4c** established a hydrogen bond with Glu304 via hydroxy group, two π-cation interactions with His279 and His239 via coumarin and indole rings, respectively, and a π-anion interaction with Glu304 via pendant 4-methoxyphenyl group. This compound also formed three none-classical hydrogen bonds with Pro309, Thr307, and Glu304, and two hydrophobic interactions with Pro309 and Arg312, and an unfavorable interaction with Arg312.

### In silico druglikeness, ADME, and toxicity studies

Druglikeness, ADME, and toxicity prediction of the most potent compounds **4a-d**, **4g**, and **4m** were performed using by online software PreADMET [32]. The obtained results were showed in Table 2. This table demonstrated that all title compounds followed of Lipinski ‘Rule of five’. Therefore, presumably, compounds **4a–d**, **4g**, and **4m** are orally active. These compounds have moderate (**4a–d**) to poor (**4g** and **4m**) permeability to Caco-2 cell. Moreover, all the studied compounds have high human intestinal absorption (HIA). Permeability of the compounds **4a–d** and **4g** to blood brain barrier (BBB) is not in the acceptable range while permeability of compound **4m** to BBB is in the acceptable range. Skin permeability of all the title compounds is in the acceptable range. All the studied compounds, with the exception of compound **4d**, are mutagenic. Compounds **4a–d**, **4g**, and **4m** have not carcinogenic effect on mouse. Moreover, compounds **4a-c** and **4g** have not carcinogenic effect on rat while compounds **4d** and **4m** are carcinogen on rat. In term of cardiotoxicity (hERG inhibition), all the title compounds have high risk.

**Table 2:** Druglikeness/ADMET prediction of the most potent compounds **4a**–**d**, **4g**, and **4m**

Druglikeness/ADME^a^/T	Compound					
	**4a**	**4b**	**4c**	**4d**	**4g**	**4m**
Rule of Five^b^	Suitable	Suitable	Suitable	Suitable	Suitable	Suitable
Caco2	29.7795	28.7663	40.7159	33.0844	23.5917	21.1337
HIA	93.264991	93.395854	93.287718	94.203919	93.27596	92.47756
BBB	6.36078	7.14551	4.50444	7.41116	6.80879	0.948237
Skin_Permeability	− 3.39577	− 3.20105	− 3.36115	− 2.65212	− 3.61849	− 3.41006
Ames_test	Mutagen	Mutagen	Mutagen	Non-mutagen	Mutagen	Mutagen
Carcino_Mouse	Negative	Negative	Negative	Negative	Negative	Negative
Carcino_Rat	Negative	Negative	Negative	Positive	Negative	Positive
hERG_inhibition	High risk	High risk	High risk	High risk	High risk	High risk

^a^The recommended ranges for Caco2: < 25 poor, > 500 great, HIA: > 80% is high < 25% is poor, BBB = − 3.0 to 1.2, and Skin_Permeability = − 8.0 to − 1.0

^b^ MW ≤ 500, HBD ≤ 5, HBA ≤ 10 and Clog P ≤ 5

## Experimental

### General procedure for the preparation of coumarin-indole derivatives 4a–m

A mixture of 4-hydroxycoumarin **1** (1.0 mmol), benzaldehyde derivatives **2a–m** (1.0 mmol), and 1*H*-indole **3** (1.0 mmol) was heated at 50 °C for 24 h in solvent free condition. After that, the mixture was washed with petroleum ether and the obtained participate was purified using recrystallization from ethyl acetate to obtain pure products **4a–m**.

#### 3-((1*H*-indol-3-yl)(phenyl)methyl)-4-hydroxy-2*H*-chromen-2-one (4a)

Isolated yield: 87%, mp: 231–233 °C; IR (KBr) 3518, 1740, 1401, 1271, 1142 cm^−1^; ^1^H NMR (300 MHz, DMSO-*d*_6_) δ 11.65 (s, 1H), 10.95 (s, 1H), 8.05 (d, *J* = 8.2 Hz, 1H), 7.61 (td, *J* = 7.9, 7.2, 1.5 Hz, 1H), 7.43–7.31 (m, 6H), 7.27 (t, *J* = 7.3 Hz, 2H), 7.22–7.12 (m, 2H), 7.12–7.02 (m, 1H), 6.98–6.89 (m, 1H), 6.15 (s, 1H). ^13^C NMR (75 MHz, DMSO-*d*_6_) δ 162.24, 160.76, 152.65, 143.18, 136.46, 132.34, 128.63, 128.19, 127.77, 126.15, 124.81, 124.26, 123.92, 121.28, 118.93, 118.78, 116.75, 116.66, 114.64, 111.93, 109.00, 37.56 ppm. MS (EI): 367.1 m/z. Anal. Calcd. for C_24_H_17_NO_3_: C, 78.46; H, 4.66; N, 3.81. Found: C, 78.65; H, 4.81; N, 3.62.

#### 3-((1*H*-indol-3-yl)(p-tolyl)methyl)-4-hydroxy-2*H*-chromen-2-one (4b)

Isolated yield: 79%, mp: 269–271 °C; IR (KBr) 3397, 1737, 1387, 1284, 1122 cm^−1^; ^1^H NMR (500 MHz, DMSO-*d*_6_) δ 9.69 (s, 2H), 7.89 (d, *J* = 7.9 Hz, 2H), 7.63–7.52 (m, 2H), 7.42–7.24 (m, 4H), 7.02 (s, 5H), 6.31 (s, 1H), 2.23 (s, 3H). ^13^C NMR (125 MHz, DMSO-*d*_6_) δ 165.63, 165.26, 152.63, 137.14, 134.87, 132.31, 129.11, 128.73, 128.52, 127.06, 124.32, 124.18, 123.89, 121.11, 118.36, 118.30, 116.39, 116.31, 114.23, 111.86, 104.69, 36.06, 20.97 ppm. MS (EI): 381.1 m/z. Anal. Calcd. for C_25_H_19_NO_3_: C, 78.72; H, 5.02; N, 3.67. Found: C, 78.95; H, 5.19; N, 3.37.

#### 3-((1*H*-indol-3-yl)(4-methoxyphenyl)methyl)-4-hydroxy-2*H*-chromen-2-one (4c)

Isolated yield: 85%, mp: 246–248 °C; IR (KBr) 3406, 1736, 1387, 1243, 1123 cm^−1^; ^1^H NMR (500 MHz, DMSO-*d*_6_) δ 11.86 (s, 2H), 7.90 (d, *J* = 8.0 Hz, 2H), 7.65–7.55 (m, 2H), 7.44–7.23 (m, 5H), 7.05 (d, *J* = 8.6 Hz, 2H), 6.79 (d, *J* = 7.2 Hz, 2H), 6.29 (s, 1H), 3.69 (s, 3H). ^13^C NMR (125 MHz, DMSO-*d*_6_) δ 165.40, 165.25, 157.79, 152.60, 133.13, 132.37, 131.73, 128.21, 124.35, 124.30, 124.23, 123.63, 121.52, 118.22, 118.11, 116.80, 116.42, 114.89, 113.95, 111.99, 104.86, 55.40, 35.69 ppm. MS (EI): 397.1 m/z. Anal. Calcd. for C_25_H_19_NO_4_: C, 75.55; H, 4.82; N, 3.52. Found: C, 75.83; H, 5.03; N, 3.29.

#### 3-((1*H*-indol-3-yl)(3-phenoxyphenyl)methyl)-4-hydroxy-2*H*-chromen-2-one (4d)

Isolated yield: 83%, mp: 183–185 °C; IR (KBr) 3489, 1729, 1401, 1231, 1112 cm^−1^; ^1^H NMR (500 MHz, DMSO-*d*_6_) δ 10.89 (s, 2H), 7.88 (dd, *J* = 7.9, 1.6 Hz, 2H), 7.61–7.52 (m, 2H), 7.26 (dq, *J* = 37.6, 8.2 Hz, 9H), 6.94 (dq, *J* = 32.0, 6.5, 5.7 Hz, 3H), 6.82 (s, 1H), 6.75 (dd, *J* = 8.0, 2.2 Hz, 1H), 6.31 (s, 1H). ^13^C NMR (125 MHz, DMSO-*d*_6_) δ 166.45, 165.08, 157.21, 156.53, 152.73, 143.55, 136.25, 132.12, 130.25, 130.19, 129.92, 127.25, 124.41, 123.96, 123.92, 123.32, 122.48, 121.27, 118.84, 118.38, 117.96, 116.27, 116.19, 114.08, 111.91, 109.99, 104.18, 36.49 ppm. MS (EI): 459.1 m/z. Anal. Calcd. for C_30_H_21_NO_4_: C, 78.42; H, 4.61; N, 3.05. Found: C, 78.25; H, 4.36; N, 3.24.

#### 4-Hydroxy-3-((3-hydroxyphenyl)(1*H*-indol-3-yl)methyl)-2*H*-chromen-2-one (4e)

Isolated yield: 80%, mp: 200–202 °C; IR (KBr) 3509, 1735, 1399, 1226, 1110 cm^−1^; ^1^H NMR (500 MHz, DMSO-*d*_6_) δ 8.04 (s, 3H), 7.90 (dd, *J* = 8.0, 1.8 Hz, 2H), 7.68–7.53 (m, 2H), 7.41 – 7.26 (m, 4H), 7.00 (t, *J* = 7.5 Hz, 1H), 6.61–6.45 (m, 3H), 6.28 (s, 1H). ^13^C NMR (125 MHz, DMSO-*d*_6_) δ 165.73, 165.26, 157.67, 152.62, 141.85, 136.51, 132.35, 129.42, 126.68, 124.37, 124.21, 123.98, 121.22, 118.37, 118.34, 117.85, 116.42, 116.39, 114.03, 113.05, 109.95, 104.55, 36.28 ppm. MS (EI): 383.4 m/z. Anal. Calcd. for C_24_H_17_NO_4_: C, 75.19; H, 4.47; N, 3.65. Found: C, 74.93; H, 4.69; N, 3.81.

#### 4-Hydroxy-3-((4-hydroxyphenyl)(1*H*-indol-3-yl)methyl)-2*H*-chromen-2-one (4f)

Isolated yield: 86%, mp: 286–288 °C; IR (KBr) 3513, 1732, 1378, 1241, 1089 cm^−1^; ^1^H NMR (300 MHz, DMSO-*d*_6_) δ 12.88 (s, 3H), 7.82 (dd, *J* = 7.9, 1.6 Hz, 2H), 7.50 (ddd, *J* = 8.6, 7.2, 1.7 Hz, 2H), 7.30–7.14 (m, 5H), 7.08 (d, *J* = 8.6 Hz, 2H), 6.56 (d, *J* = 8.6 Hz, 2H), 6.17 (s, 1H). ^13^C NMR (75 MHz, DMSO-*d*_6_) δ 168.00, 165.01, 155.08, 152.94, 140.00, 132.78, 131.21, 128.00, 124.55, 124.36, 123.68, 123.25, 121.45, 118.39, 118.27, 117.06, 116.85, 114.95, 114.70, 112.02, 104.28, 35.79 ppm. MS (EI): 383.2 m/z. Anal. Calcd. for C_24_H_17_NO_4_: C, 75.19; H, 4.47; N, 3.65. Found: C, 75.38; H, 4.57; N, 3.39.

#### 3-((3-Fluorophenyl)(1*H*-indol-3-yl)methyl)-4-hydroxy-2*H*-chromen-2-one (4g)

Isolated yield: 87%, mp: 222–224 °C; IR (KBr) 3494, 1730, 1410, 1259, 1126 cm^−1^; ^1^H NMR (300 MHz, DMSO-*d*_6_) δ 11.78 (s, 1H), 11.00 (s, 1H), 8.06 (d, *J* = 8.2 Hz, 1H), 7.63 (ddd, *J* = 8.6, 7.1, 1.5 Hz, 1H), 7.45–7.30 (m, 5H), 7.22–7.14 (m, 2H), 7.12–6.84 (m, 4H), 6.15 (s, 1H). ^13^C NMR (75 MHz, DMSO-*d*_6_) δ 164.07 (^1^*J*_CF_ = 240 Hz), 162.26, 161.02, 152.69, 146.51, 136.45 (^3^*J*_CF_ = 6.75 Hz), 132.45, 130.01 (^3^*J*_CF_ = 8.25 Hz), 127.59, 124.86, 124.68 (^4^*J*_CF_ = 2.25 Hz), 124.29, 123.99, 121.39, 118.92, 118.87, 116.85, 116.71, 115.49 (^2^*J*_CF_ = 21.75 Hz), 113.95, 113.07 (^2^*J*_CF_ = 21 Hz), 112.01, 108.57, 37.30 ppm. MS (EI): 385.3 m/z. Anal. Calcd. for C_24_H_16_FNO_3_: C, 74.80; H, 4.18; N, 3.63. Found: C, 75.05; H, 3.96; N, 3.77.

#### 3-((4-Fluorophenyl)(1*H*-indol-3-yl)methyl)-4-hydroxy-2*H*-chromen-2-one (4h)

Isolated yield: 85%, mp: 243–245 °C; IR (KBr) 3490, 1736, 1392, 1237, 1073, 912 cm^−1^; ^1^H NMR (500 MHz, DMSO-*d*_6_) δ 11.65 (s, 1H), 10.92 (s, 1H), 8.03 (d, *J* = 7.9 Hz, 1H), 7.58 (t, *J* = 7.8 Hz, 1H), 7.45 – 7.18 (m, 6H), 7.11 (d, *J* = 2.4 Hz, 1H), 7.04 (dd, *J* = 10.3, 7.8 Hz, 3H), 6.90 (t, *J* = 7.5 Hz, 1H), 6.10 (s, 1H). ^13^C NMR (125 MHz, DMSO-*d*_6_) δ 162.18, 161.93 (^1^*J*_CF_ = 240 Hz), 160.78, 152.64, 139.22 (^4^*J*_CF_ = 2.5 Hz), 136.49, 132.34, 130.43 (^3^*J*_CF_ = 7.5 Hz), 127.56, 124.72, 124.23, 123.93, 121.30, 118.89, 118.81, 116.70, 116.64, 114.82 (^2^*J*_CF_ = 20 Hz), 114.51, 111.93, 108.82, 36.94 ppm. MS (EI): 385.1 m/z. Anal. Calcd. for C_24_H_16_FNO_3_: C, 74.80; H, 4.18; N, 3.63. Found: C, 74.62; H, 4.41; N, 3.36.

#### 3-((3-Chlorophenyl)(1*H*-indol-3-yl)methyl)-4-hydroxy-2*H*-chromen-2-one (4i)

Isolated yield: 80%, mp: 205–207 °C; IR (KBr) 3513, 1728, 1354, 1270, 1118 cm^−1^; ^1^H NMR (300 MHz, DMSO-*d*_6_) δ 12.21 (s, 1H), 11.00 (s, 1H), 8.06 (d, *J* = 8.0 Hz, 1H), 7.84 (dd, *J* = 7.8, 1.4 Hz, 1H), 7.75–7.53 (m, 2H), 7.45–7.32 (m, 6H), 7.20 (d, *J* = 2.1 Hz, 1H), 7.09 (t, *J* = 7.2 Hz, 1H), 6.95 (t, *J* = 7.4 Hz, 1H), 6.15 (s, 1H). ^13^C NMR (75 MHz, DMSO-*d*_6_) δ 162.29, 161.15, 152.71, 136.46, 133.13, 132.90, 132.43, 130.02, 128.37, 127.56, 127.38, 126.15, 124.88, 124.36, 123.67, 121.43, 118.95, 118.88, 116.75, 116.70, 113.81, 112.03, 108.47, 37.27 ppm. MS (EI): 401.0 m/z. Anal. Calcd. for C_24_H_16_ClNO_3_: C, 71.73; H, 4.01; N, 3.49. Found: C, 71.98; H, 4.16; N, 3.27.

#### 3-((4-Chlorophenyl)(1*H*-indol-3-yl)methyl)-4-hydroxy-2*H*-chromen-2-one (4j)

Isolated yield: 82%, mp: 235–237 °C; IR (KBr) 3482, 1663, 1727, 1411, 1240, 1100 cm^−1^; ^1^H NMR (499 MHz, DMSO-*d*_6_) δ 11.71 (s, 1H), 10.95 (s, 1H), 8.04 (d, *J* = 7.8 Hz, 1H), 7.61 (td, *J* = 7.6, 7.0, 1.5 Hz, 1H), 7.41–7.22 (m, 8H), 7.14 (s, 1H), 7.06 (t, *J* = 7.4 Hz, 1H), 6.92 (t, *J* = 7.4 Hz, 1H), 6.09 (s, 1H). ^13^C NMR (126 MHz, DMSO-*d*_6_) δ 162.26, 161.67, 152.58, 140.06, 136.57, 133.36, 132.16, 130.86, 127.22, 125.50, 124.69, 124.49, 123.89, 121.31, 118.69, 118.59, 116.72, 116.61, 114.32, 111.78, 104.82, 35.77 ppm. MS (EI): 401.0 m/z. Anal. Calcd. for C_24_H_16_ClNO_3_: C, 71.73; H, 4.01; N, 3.49. Found: C, 71.56; H, 3.87; N, 3.70.

#### 3-((3-Bromophenyl)(1*H*-indol-3-yl)methyl)-4-hydroxy-2*H*-chromen-2-one (4k)

Isolated yield: 86%, mp: 212–214 °C; IR (KBr) 3505, 1737, 1412, 1272, 1010 cm^−1^; ^1^H NMR (500 MHz, DMSO-*d*_6_) δ 11.74 (s, 1H), 10.95 (s, 1H), 8.02 (d, *J* = 7.9 Hz, 1H), 7.60 (t, *J* = 7.7 Hz, 1H), 7.40–7.26 (m, 7H), 7.20 (t, *J* = 7.9 Hz, 1H), 7.13 (d, *J* = 2.4 Hz, 1H), 7.05 (t, *J* = 7.6 Hz, 1H), 6.91 (t, *J* = 7.5 Hz, 1H), 6.09 (s, 1H). ^13^C NMR (125 MHz, DMSO-*d*_6_) δ 162.21, 161.01, 152.66, 146.24, 136.41, 132.46, 131.15, 130.36, 129.03, 127.74, 127.49, 124.82, 124.29, 123.97, 121.58, 121.39, 118.91, 118.81, 116.70, 116.66, 113.71, 111.99, 108.47, 37.21 ppm. MS (EI): 445.0 m/z. Anal. Calcd. for C_24_H_16_BrNO_3_: C, 64.59; H, 3.61; N, 3.14. Found: C, 64.79; H, 3.44; N, 3.32.

#### 3-((1*H*-indol-3-yl)(2-nitrophenyl)methyl)-4-hydroxy-2*H*-chromen-2-one (4l)

Isolated yield: 84%, mp: 240–242 °C; IR (KBr) 3488, 1726, 1551, 1357, 1239, 1101 cm−^1^; ^1^H NMR (500 MHz, DMSO-*d*_6_) δ 11.71 (s, 2H), 7.84 (d, *J* = 7.9 Hz, 2H), 7.65 (d, *J* = 7.9 Hz, 1H), 7.59 – 7.46 (m, 4H), 7.40 (d, *J* = 7.8 Hz, 3H), 7.34–7.19 (m, 5H), 6.52 (s, 1H). ^13^C NMR (125 MHz, DMSO-*d*_6_) δ 166.02, 163.74, 152.76, 149.92, 135.27, 132.32, 132.03, 130.28, 128.97, 127.42, 126.36, 124.42, 124.26, 123.93, 123.85, 121.70, 118.69, 118.52, 116.43, 116.30, 114.87, 112.00, 103.65, 34.67 ppm. MS (EI): 412.1 m/z. Anal. Calcd. for C_24_H_16_N_2_O_5_: C, 69.90; H, 3.91; N, 6.79. Found: C, 70.17; H, 4.16; N, 6.96.

#### 3-((1*H*-indol-3-yl)(3-nitrophenyl)methyl)-4-hydroxy-2*H*-chromen-2-one (4m)

Isolated yield: 84%, mp: 198–200 °C; IR (KBr) 3521, 1729, 1557, 1353, 1150 cm^−1^; ^1^H NMR (500 MHz, DMSO-*d*_6_) δ 7.98 (d, *J* = 8.2 Hz, 1H), 7.88 (s, 1H), 7.82 (d, *J* = 7.9 Hz, 2H), 7.62–7.41 (m, 5H), 7.29 (d, *J* = 8.2 Hz, 2H), 7.24 (t, *J* = 7.6 Hz, 2H), 6.35 (s, 1H). ^13^C NMR (125 MHz, DMSO-*d*_6_) δ 167.93, 164.75, 152.96, 148.21, 145.28, 137.29, 134.30, 131.82, 129.85, 127.77, 124.58, 124.35, 123.63, 121.53, 120.86, 119.80, 118.91, 118.82, 116.12, 116.03, 114.68, 112.13, 103.22, 36.71 ppm. MS (EI): 412.1 m/z. Anal. Calcd. for C_24_H_16_N_2_O_5_: C, 69.90; H, 3.91; N, 6.79. Found: C, 70.11; H, 4.08; N, 6.58.

### In vitro α‐glucosidase inhibition assay and kinetic study

The α‐glucosidase inhibition assays of the coumarin-indole derivatives **4a–m** and kinetic study of the most potent compound **4d** were performed into yeast α‐glucosidase according to the literature [31]. α-Glucosidase (EC3.2.1.20, *Saccharomyces cerevisiae* (*S. cerevisiae*), 20 U/mg) and substrate (*p*-nitrophenyl glucopyranoside) were prepared from Sigma-Aldrich. Appropriate enzyme concentration was obtained in potassium phosphate buffer (pH 6.8, 50 mM), and coumarin-indole hybrids **4a–m** were dissolved in DMSO (10% final concentration). The potassium phosphate buffer (135 µL), various concentrations of the target compounds **4a–m** (20 µL), and prepared enzyme solution (20 µL) were added to the 96-well plate and the later mixture was incubated for 10 min at 37 °C. Then, *p*-nitrophenyl glucopyranoside (substrate, 25 µL, 4 mM) was added to the incubated mixture and allowed to incubate at 37 °C for 20 min. Finally, the change in absorbance of the final mixture was measured at 405 nm by using spectrophotometer (Gen5, Power wave xs2, BioTek, America). DMSO (10% final concentration) as negative control and acarbose as positive control were used. The percentage of enzyme inhibition (% Inhibition) for each sample was calculated by using the following formula:

$${\%\text{ Inhibition}} = [({\text{Abs control}} - {\text{Abs sample}})/{\text{Abs control}}] \times 100$$

IC_50_ values were calculated from non-linear regression curve using by the Logit method.

### Docking study

Docking study of the most potent compounds **4a–d** in the modeled α-glucosidase active site was performed according to our previously described method [31]. *S. cerevisiae* α-glucosidase that was used in the experimental section had not any crystallographic structure in the protein data bank (PDB), thus, we constructed a modeled enzyme using SWISS-MODEL Repository [33]. For this purpose, our research team used of a method that was described by Imran et al. [34, 35]. After searching by using SWISS-MODEL to identify an appropriate protein with a high sequence similarity with *S. cerevisiae* α-glucosidase in PDB, we selected *S. cerevisiae* isomaltase with PDB code of 3A4A. The latter enzyme has 72% identical and 85% similarity with the *S. cerevisiae* α-glucosidase. Next, *S. cerevisiae* isomaltase was subjected through sequence alignment and homology model using by automated homology modeling pipeline SWISS-MODEL (managed by Swiss Institute of Bioinformatics) and the quality of the obtained model was verified using PROCHECK [33].

The 3D structures of the positive control acarbose and the most potent compounds **4d**, **4a**, **4b**, and **4c** were built by MarvineSketch 5.8.3, 2012, ChemAxon (http://www.chemaxon.com) and converted to pdbqt coordinate using Auto dock Tools. The pdbqt coordinate of the modeled α-glucosidase was created using the latter software by the following process: the polar hydrogen atoms were added and the Koullman charges were assigned. The obtained pdbqt file of enzyme was used as an input file for the AUTOGRID program. In AUTOGRID for each atom type in the studied compounds, maps were calculated with 0.375 Å spacing between grid points and the center of the grid box was placed at x = 12.5825, y = − 7.8955, and z = 12.519 Å. The appropriate dimensions for the active site box were determined by BIOVIA Discovery Studio v.3.5 (40 × 40 × 40 Å). Flexible ligand dockings were accomplished for the target compounds. Each docked system for these compounds was carried out by 50 runs of the AUTODOCK search by the Lamarckian genetic algorithm. The best poses of the title compounds were selected for analyzing the interactions between enzyme and ligands. The results were visualized using BIOVIA Discovery Studio v.3.5 and the obtained data showed in Fig. 3.

### In silico druglikeness/ADME/T studies

In silico druglikeness/ADME/T prediction of the most potent compounds **4a–d**, **4g**, and **4m** were performed using the preADMET online server [32].

## Conclusion

Coumarin-indole hybrids **4a–m** considered as new α-glucosidase inhibitors and synthesized by a one-step simple reaction. Enzymatic testing of the prepared compounds exhibited that most of the title compounds are potent inhibitor against α-glucosidase and the most potent entry (compound **4d**) was a competitive inhibitor for this enzyme. SAR study of the title compounds revealed that in addition to the nature of substitution, the position of the substitutions play an important role in the observed anti-α-glucosidase activities. All the most potent compounds were docked at α-glucosidase active site. The latter study revealed that potent derivatives with coumarin-indole scaffold interacted with α-glucosidase active site with low BEs in comparison to standard inhibitor acarbose.

## Supplementary Information

**Additional file 1.** Images of ^1^H NMR and ^13^C NMR of the new synthesized compounds **5a-m** and IC_50_ graphs of these compounds are available in the Supporting Information.

## Data Availability

The datasets used or analyzed during the current study are available from the corresponding author on reasonable request.
