# Supplementary material for: Design, synthesis, in vitro, and in silico biological evaluations of coumarin-indole hybrids as new anti-α-glucosidase agents
Source: BMC Chem. 2022 Nov 3;16(1):84. doi: 10.1186/s13065-022-00882-2 (PMC9635080; doi:10.1186/s13065-022-00882-2)
Supplement: Supplementary file 1 — Additional file 1. Images of 1H NMR and 13C NMR of the new synthesized compounds 5a-m and IC50 graphs of these compounds are available in the Supporting Information. [file 13065_2022_882_MOESM1_ESM.docx]

**Support information**

**Design, synthesis, *in vitro*, and *in silico* biological evaluations of coumarin-indole hybrids as new anti-α-glucosidase agents**

Davood Rezapour Niri^1^, Mohammad Hosein Sayahi^2^, Somayeh Behrouz^1^, Ali Moazzam^3^, Somayeh Mojtabavi^4^, Mohammad Ali Faramarzi^4^, Bagher Larijani^3^, Hossein Rastegar^5^, Maryam Mohammadi-Khanaposhtani^6*^, Mohammad Mahdavi^3*^

^1^Medicinal Chemistry Research Laboratory, Department of Chemistry, Shiraz University of Technology, Shiraz, Iran. ^2^Department of Chemistry, Payame Noor University (PNU), Tehran, Iran. ^3^Endocrinology and Metabolism Research Center, Endocrinology and Metabolism Clinical Sciences Institute, Tehran University of Medical Sciences, Tehran, Iran. ^4^Department of Pharmaceutical Biotechnology, Faculty of Pharmacy, Tehran University of Medical Sciences, Tehran, Iran. ^5^Cosmetic products research center, Iranian food and drug administration, MOHE, Tehran, Iran. ^6^Cellular and Molecular Biology Research Center, Health Research Institute, Babol University of Medical Sciences, Babol, Iran.

*Correspondences:

[maryammoha@gmail.com](mailto:maryammoha@gmail.com)

Department of Pharmaceutical Biotechnology, Faculty of Pharmacy, Tehran University of Medical Sciences, Tehran, Iran

[momahdavi@tums.ac.ir](mailto:momahdavi@tums.ac.ir)

Endocrinology and Metabolism Research Center, Endocrinology and Metabolism Clinical Sciences Institute, Tehran University of Medical Sciences, Tehran, Iran

**3-((1*H*-indol-3-yl)(phenyl)methyl)-4-hydroxy-2*H*-chromen-2-one (4a)**

**
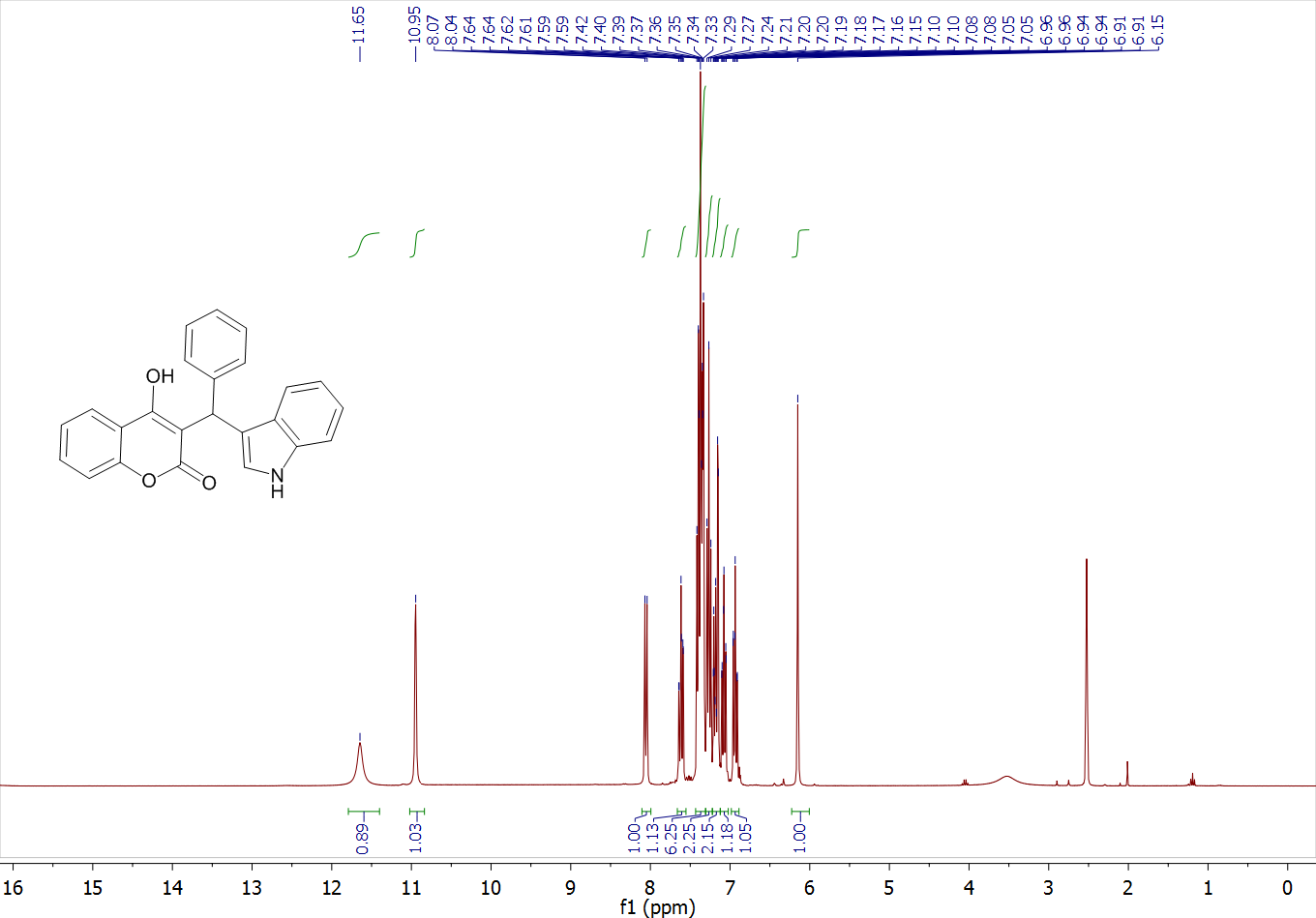
**

**
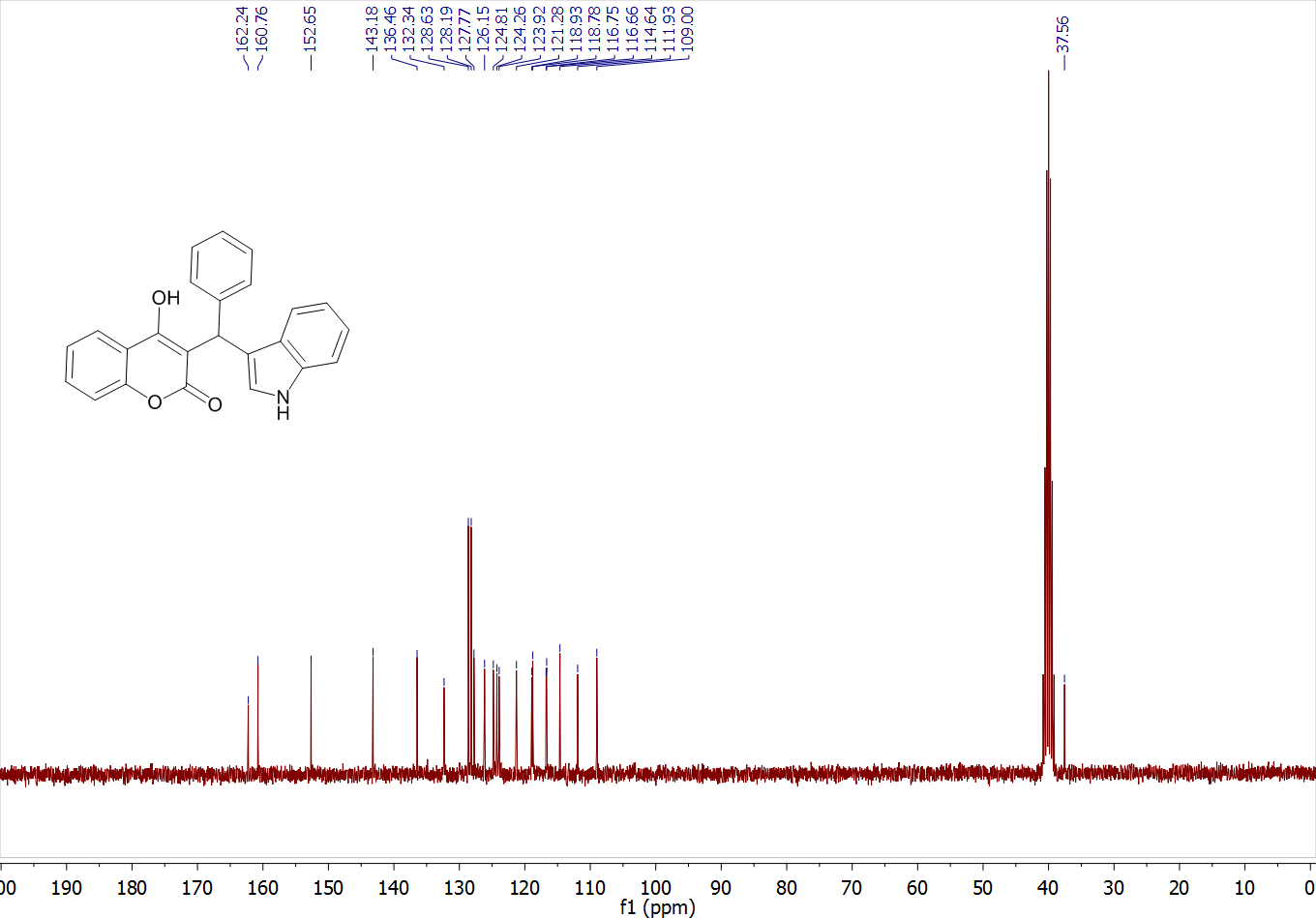
**

**3-((1*H*-indol-3-yl)(*p-*tolyl)methyl)-4-hydroxy-2*H*-chromen-2-one (4b)**

**
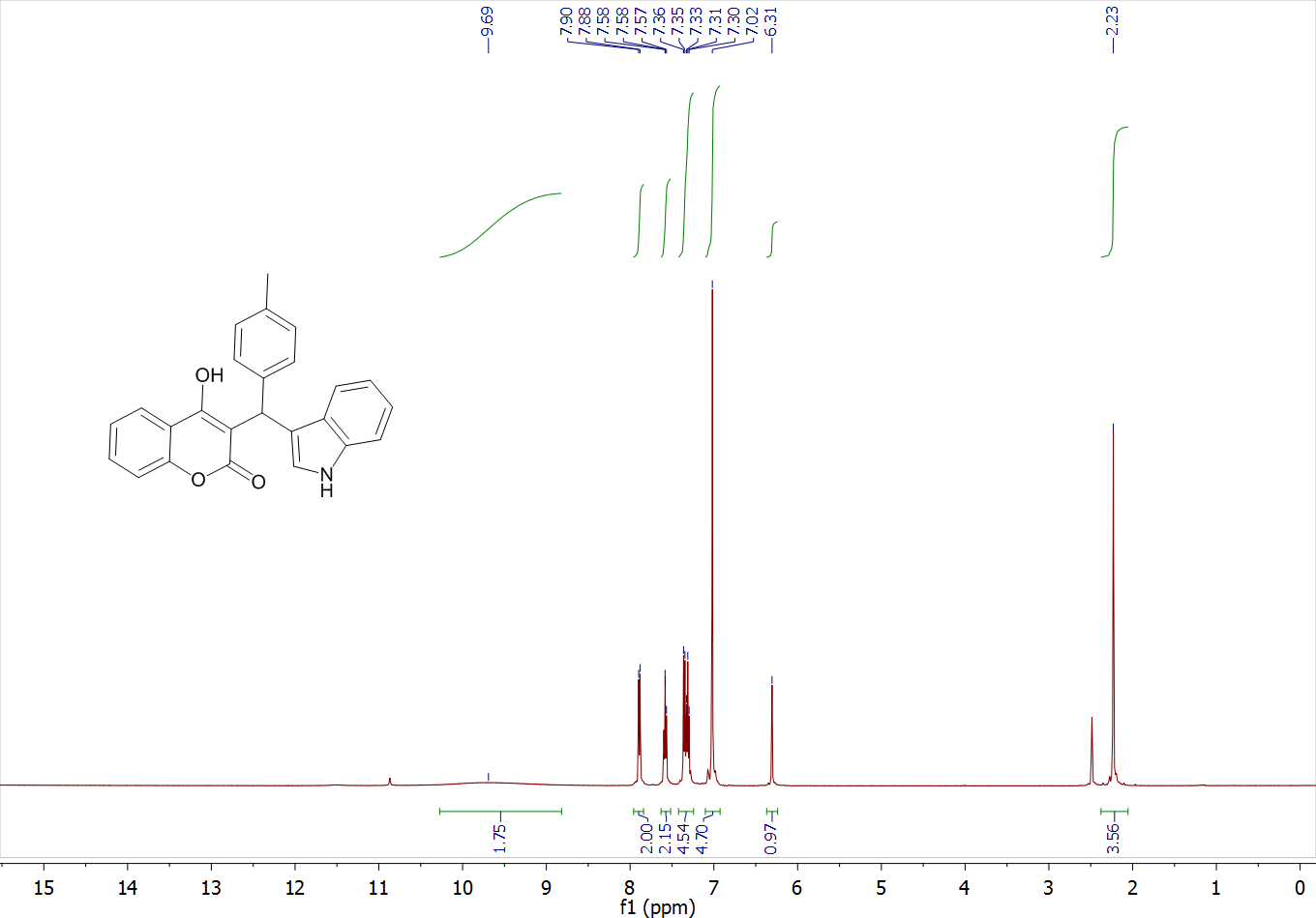
**

**
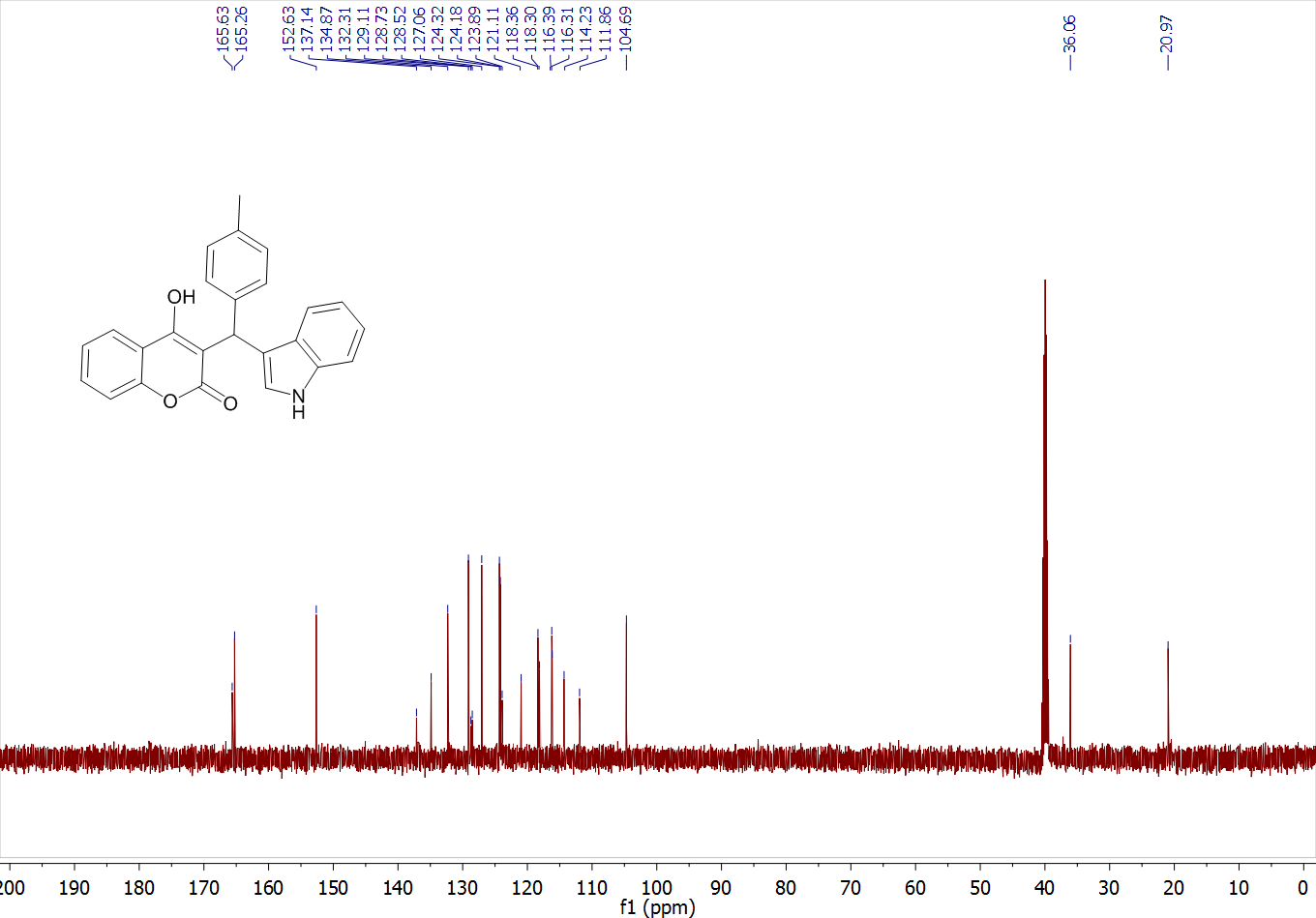
**

**3-((1*H*-indol-3-yl)(4-methoxyphenyl)methyl)-4-hydroxy-2*H*-chromen-2-one (4c)**

**
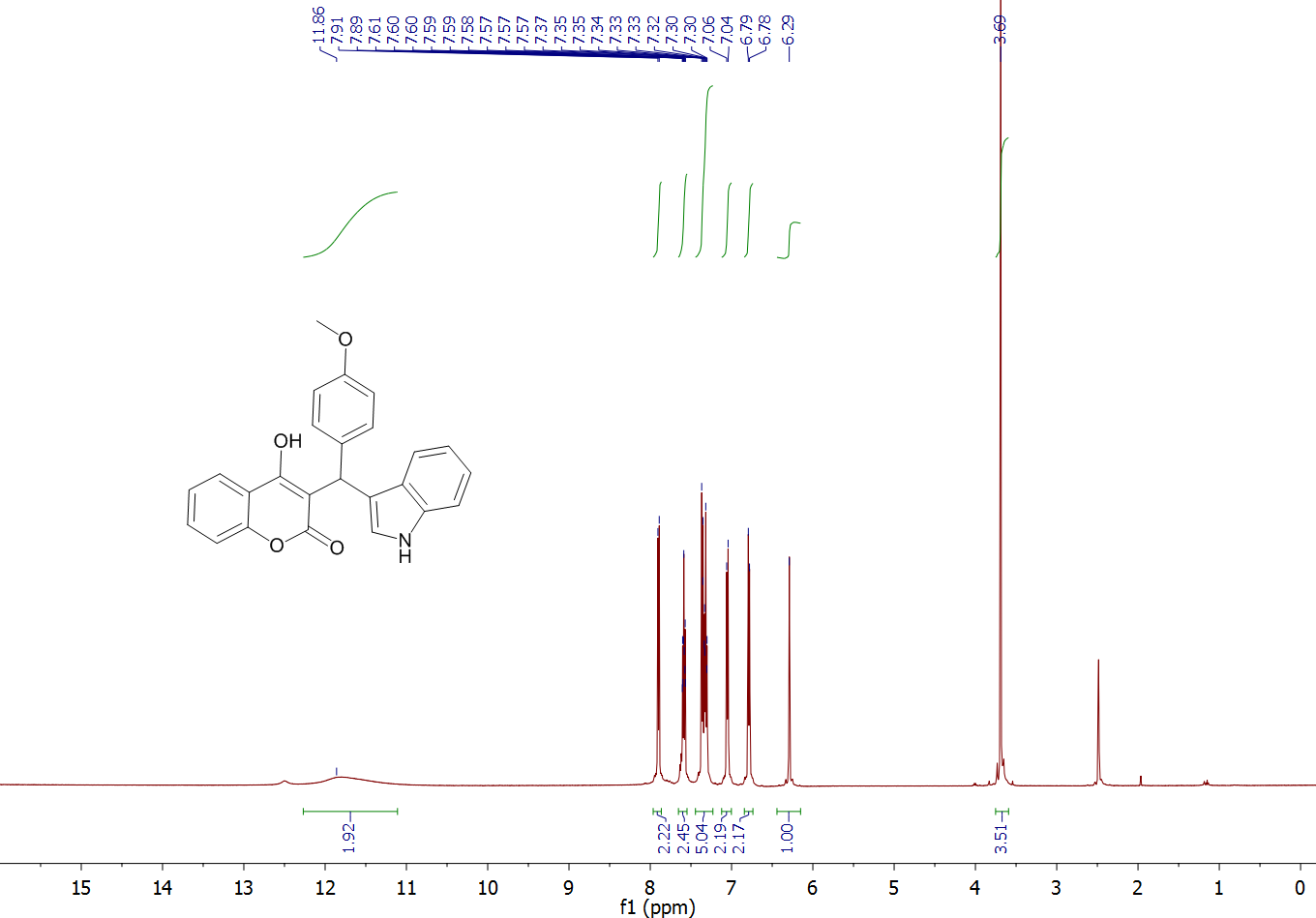
**

**
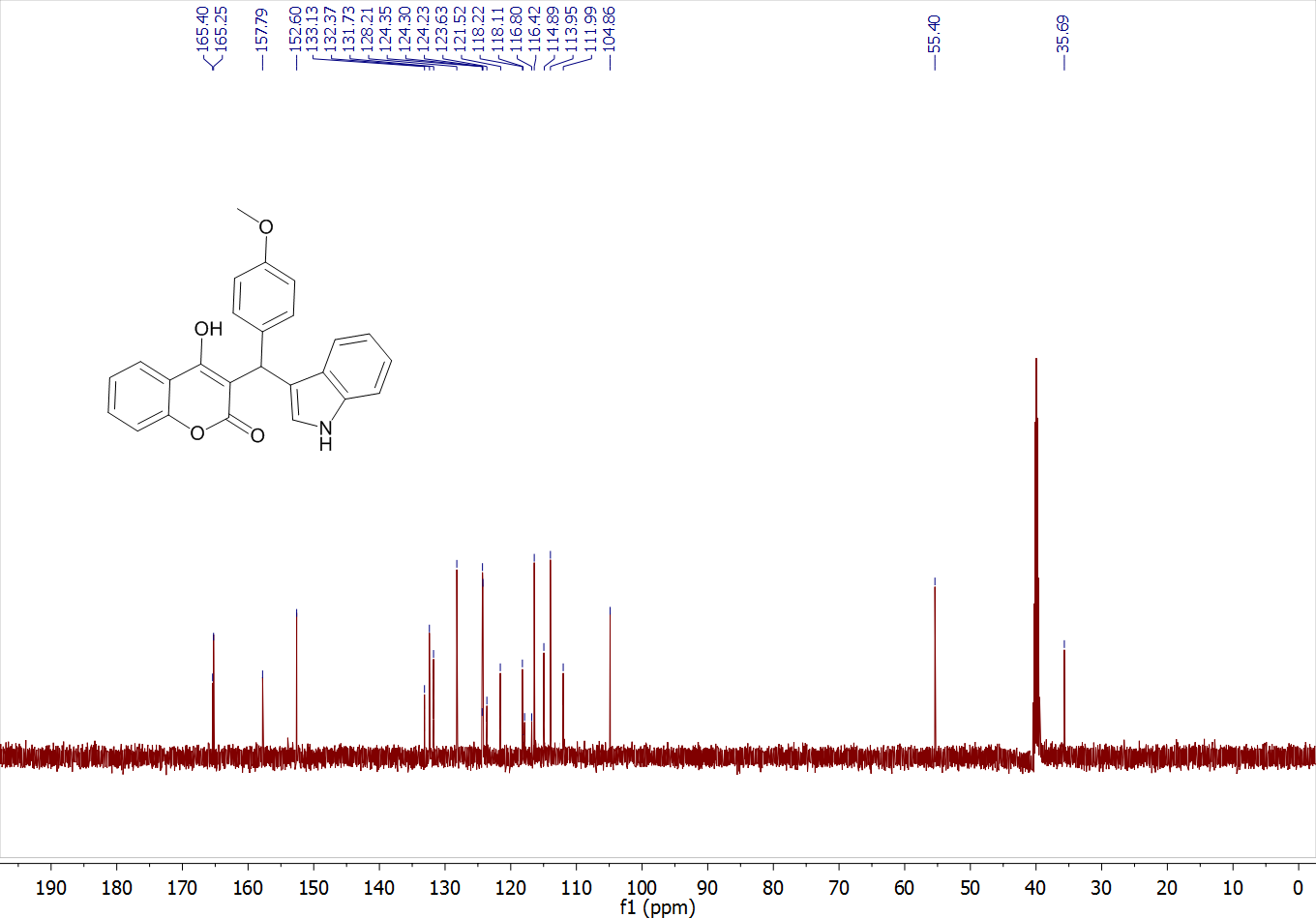
**

**3-((1*H*-indol-3-yl)(3-phenoxyphenyl)methyl)-4-hydroxy-2*H*-chromen-2-one (4d)**

**
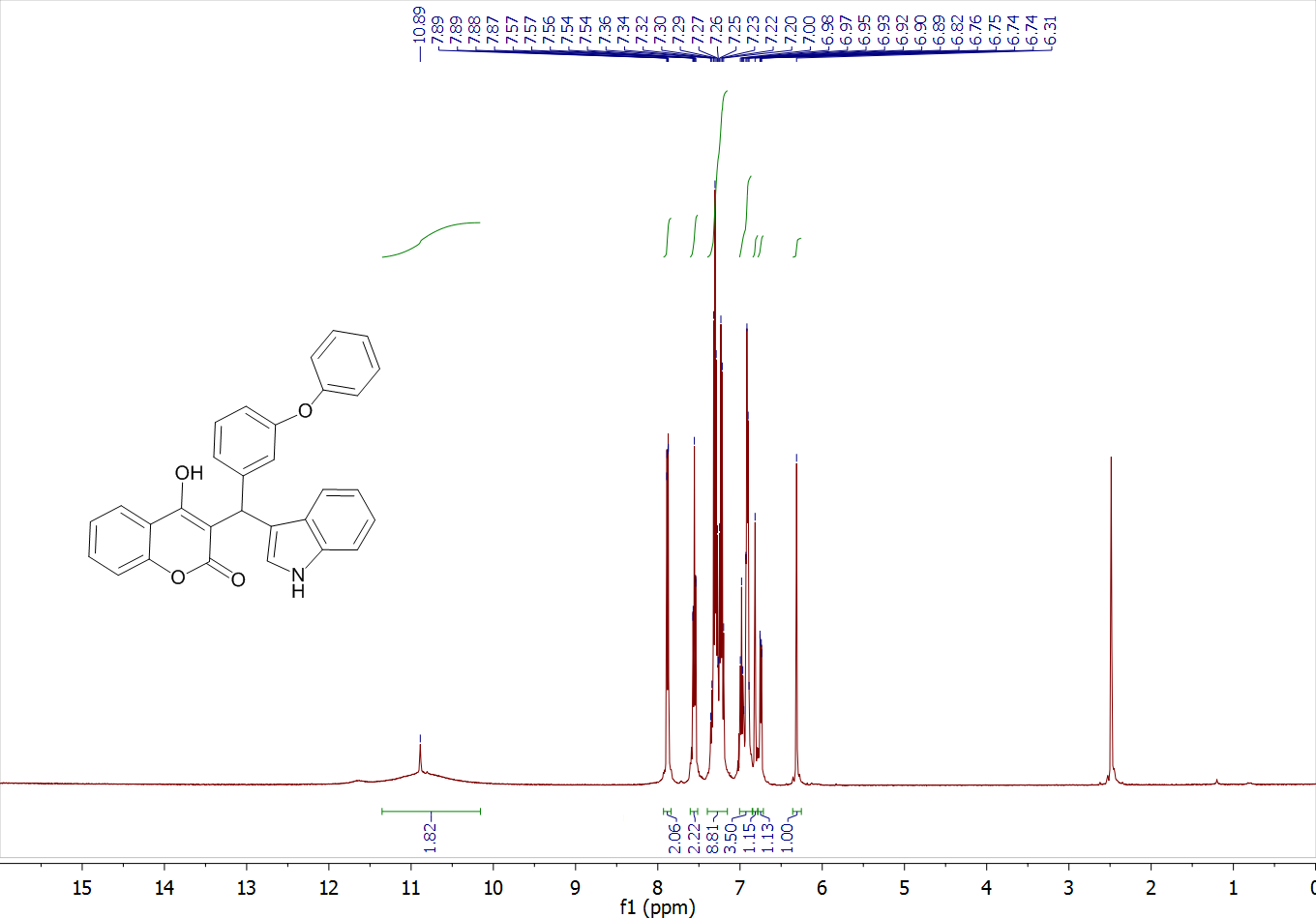
**

**
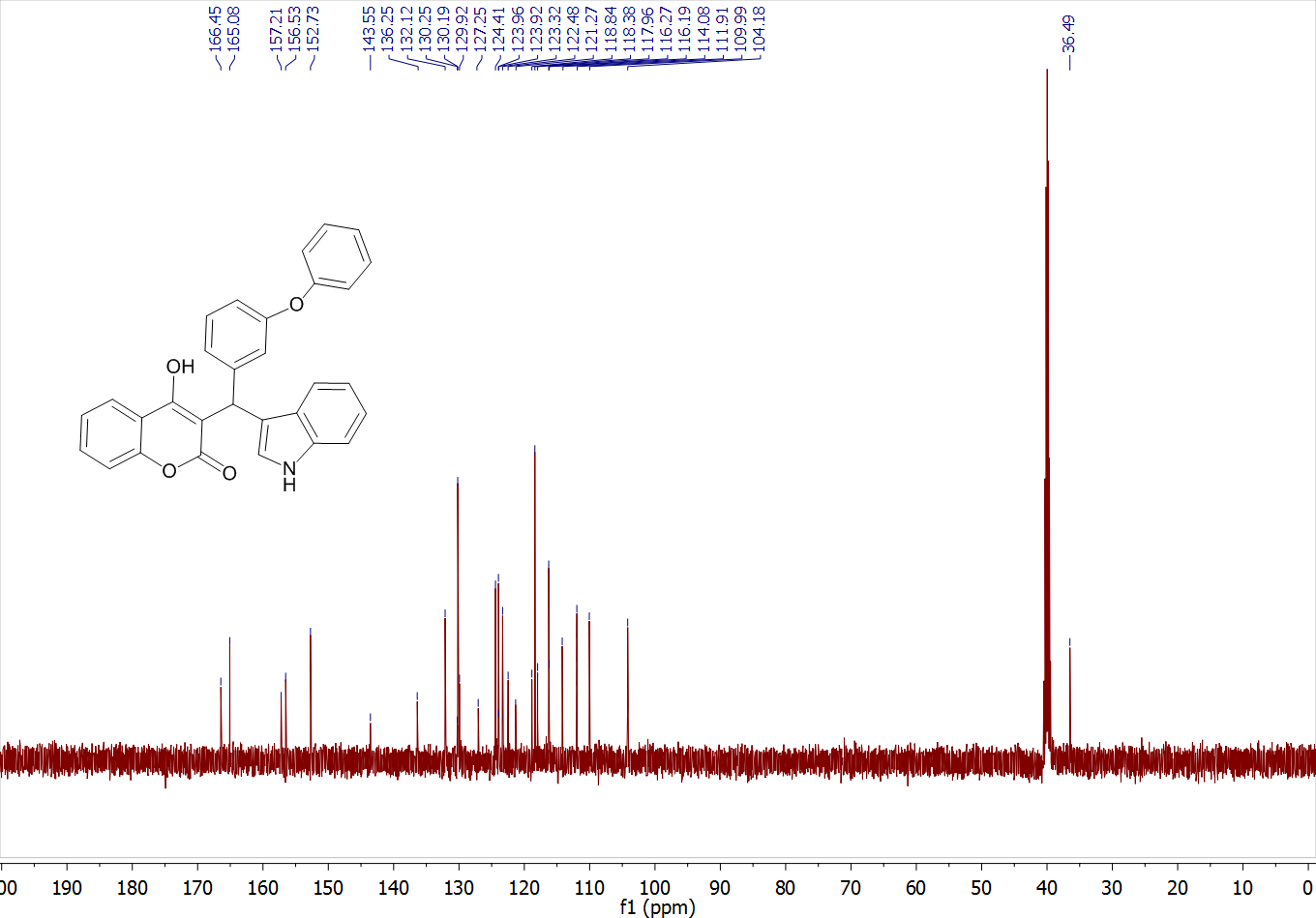
**

**4-hydroxy-3-((3-hydroxyphenyl)(1*H*-indol-3-yl)methyl)-2*H*-chromen-2-one (4e)**

**
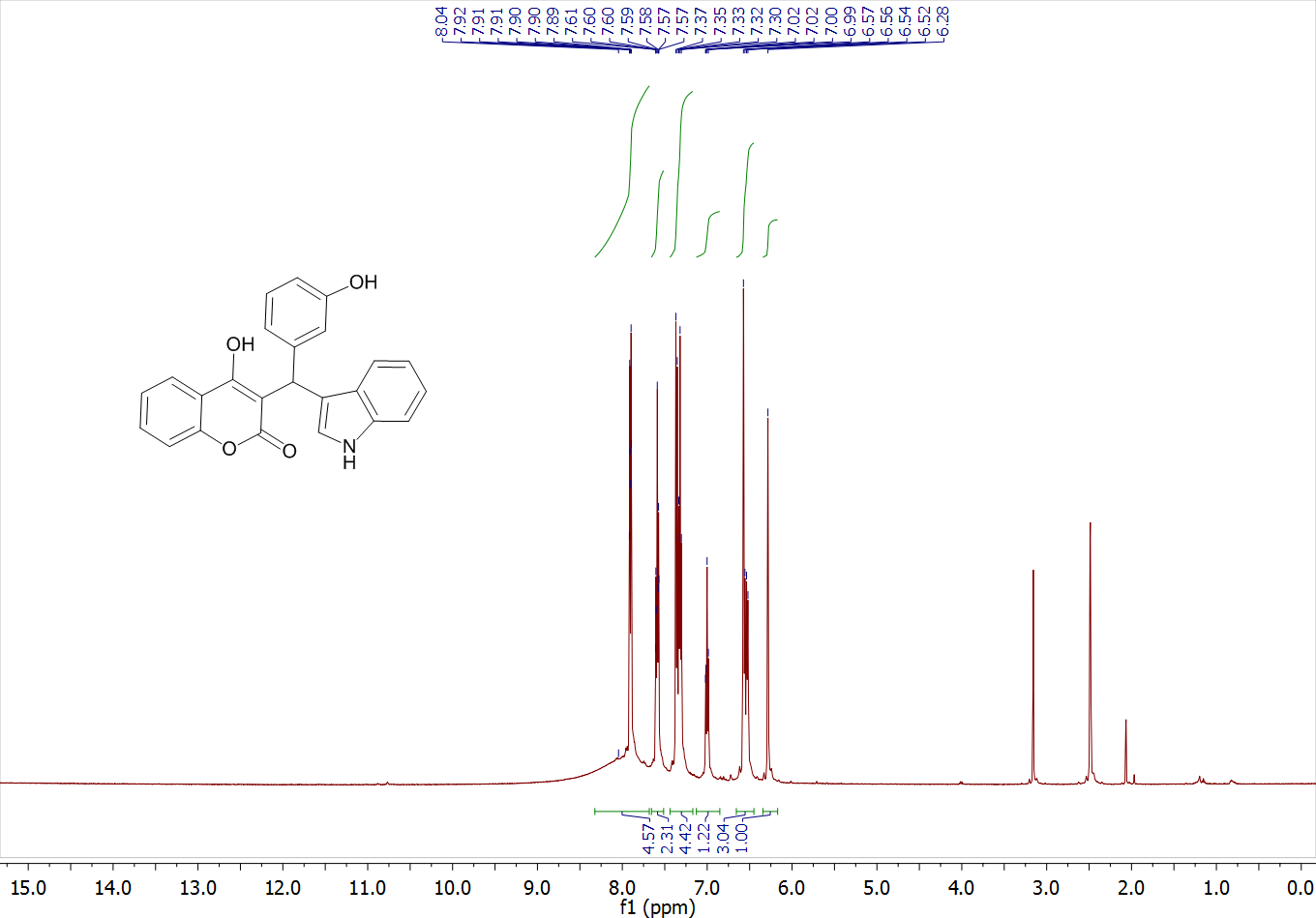
**

**
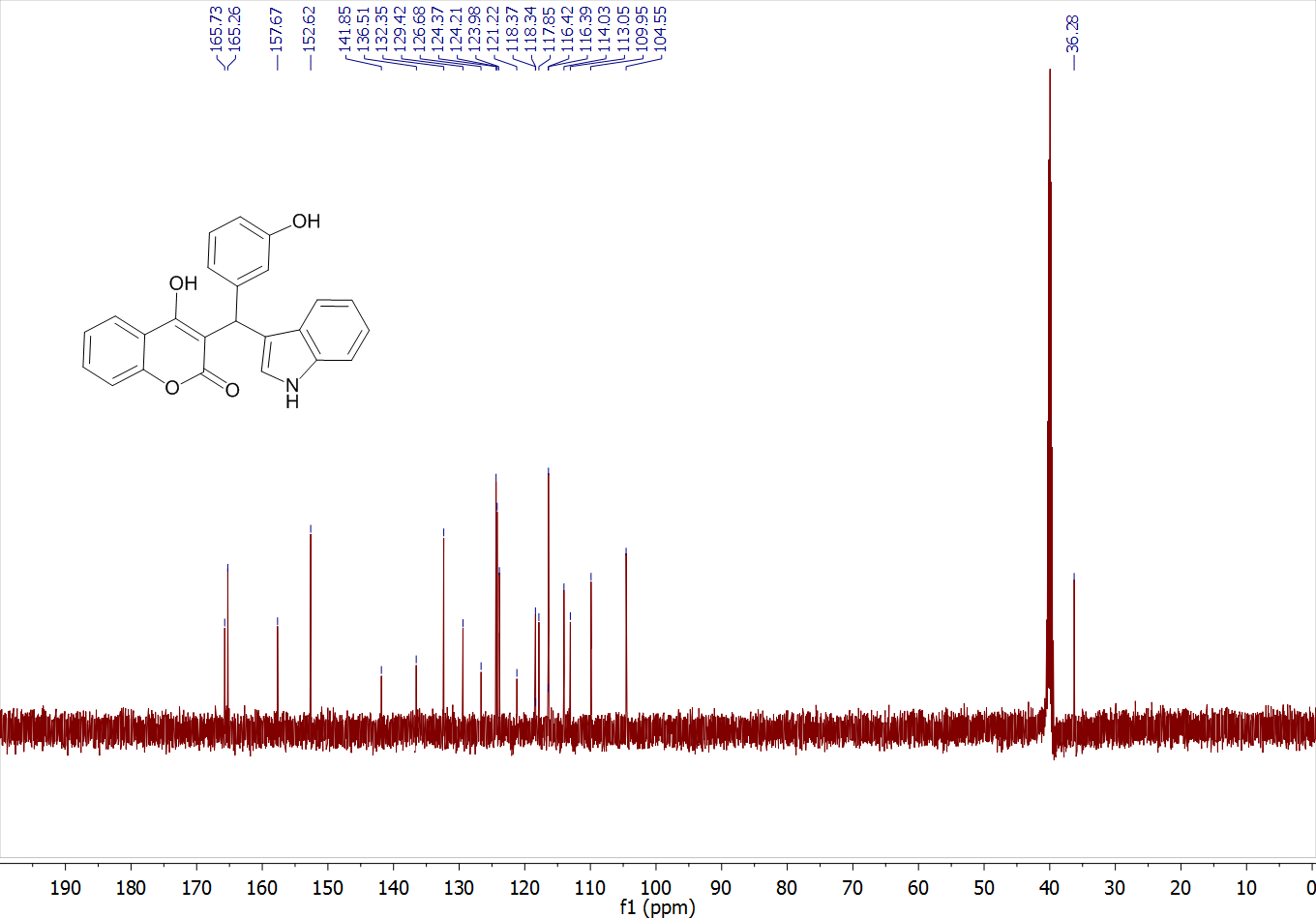
**

**4-hydroxy-3-((4-hydroxyphenyl)(1*H*-indol-3-yl)methyl)-2*H*-chromen-2-one (*4f*)**

**
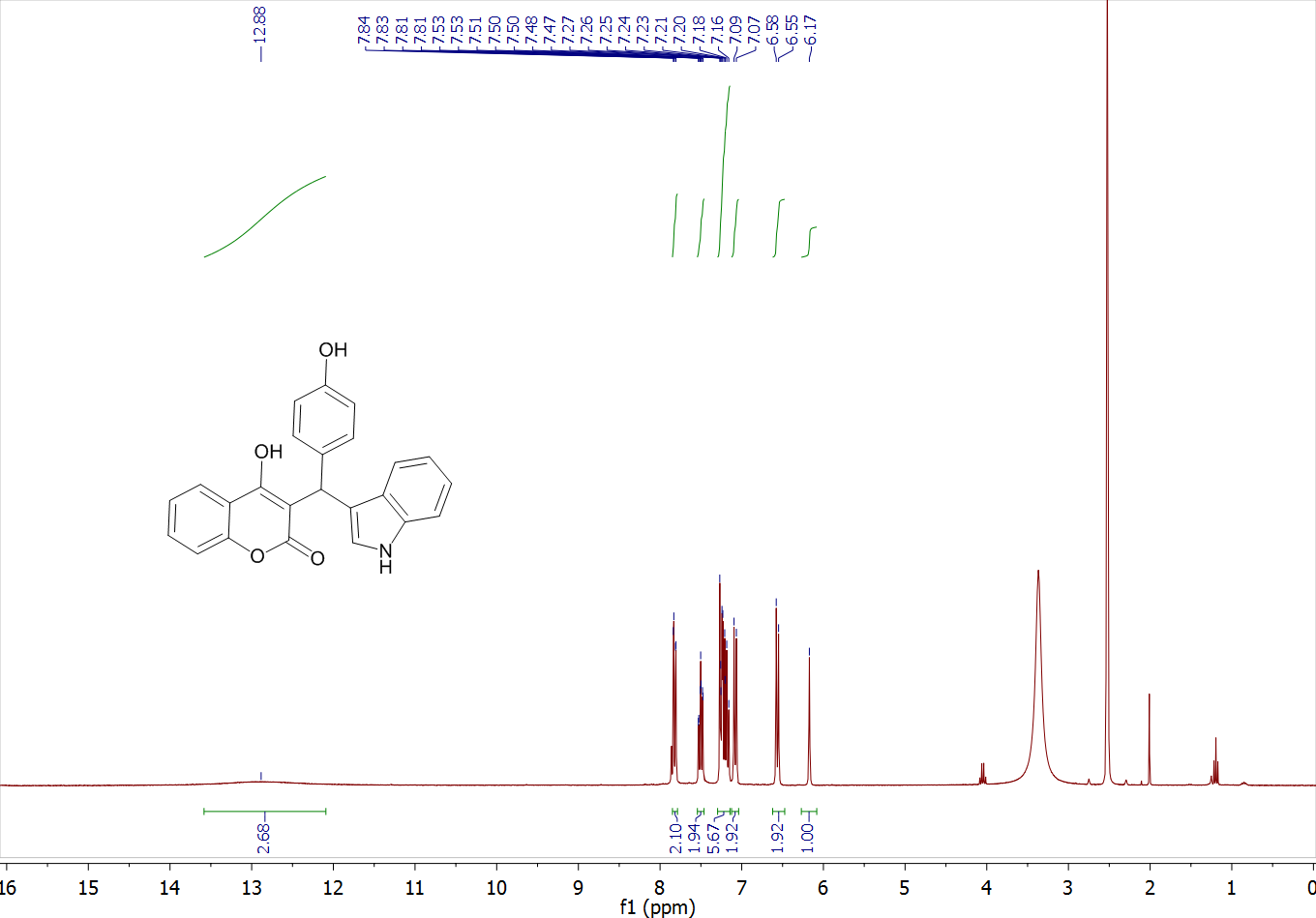
**

**
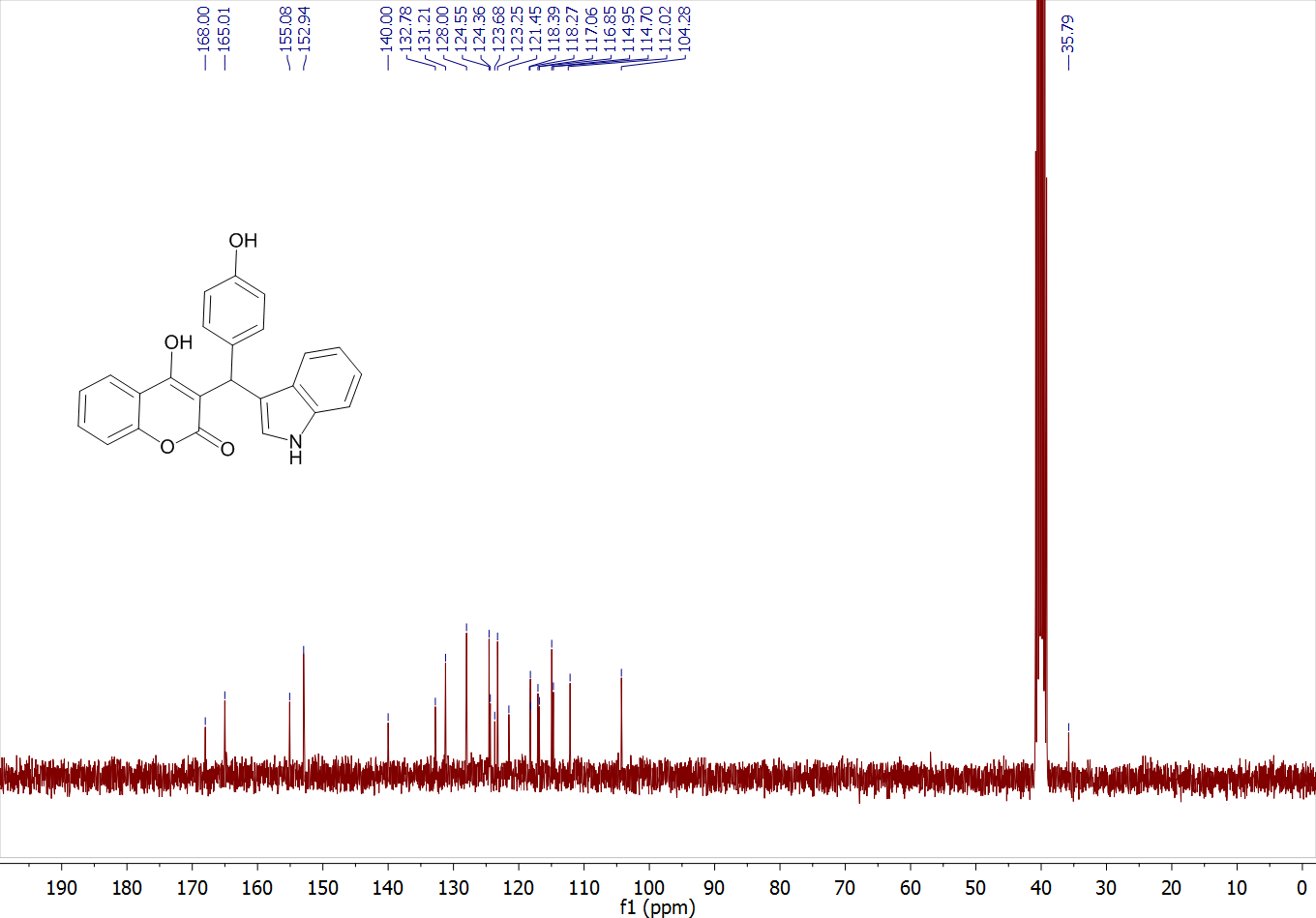
**

**3-((3-fluorophenyl)(1*H*-indol-3-yl)methyl)-4-hydroxy-2*H*-chromen-2-one (4g)**

**
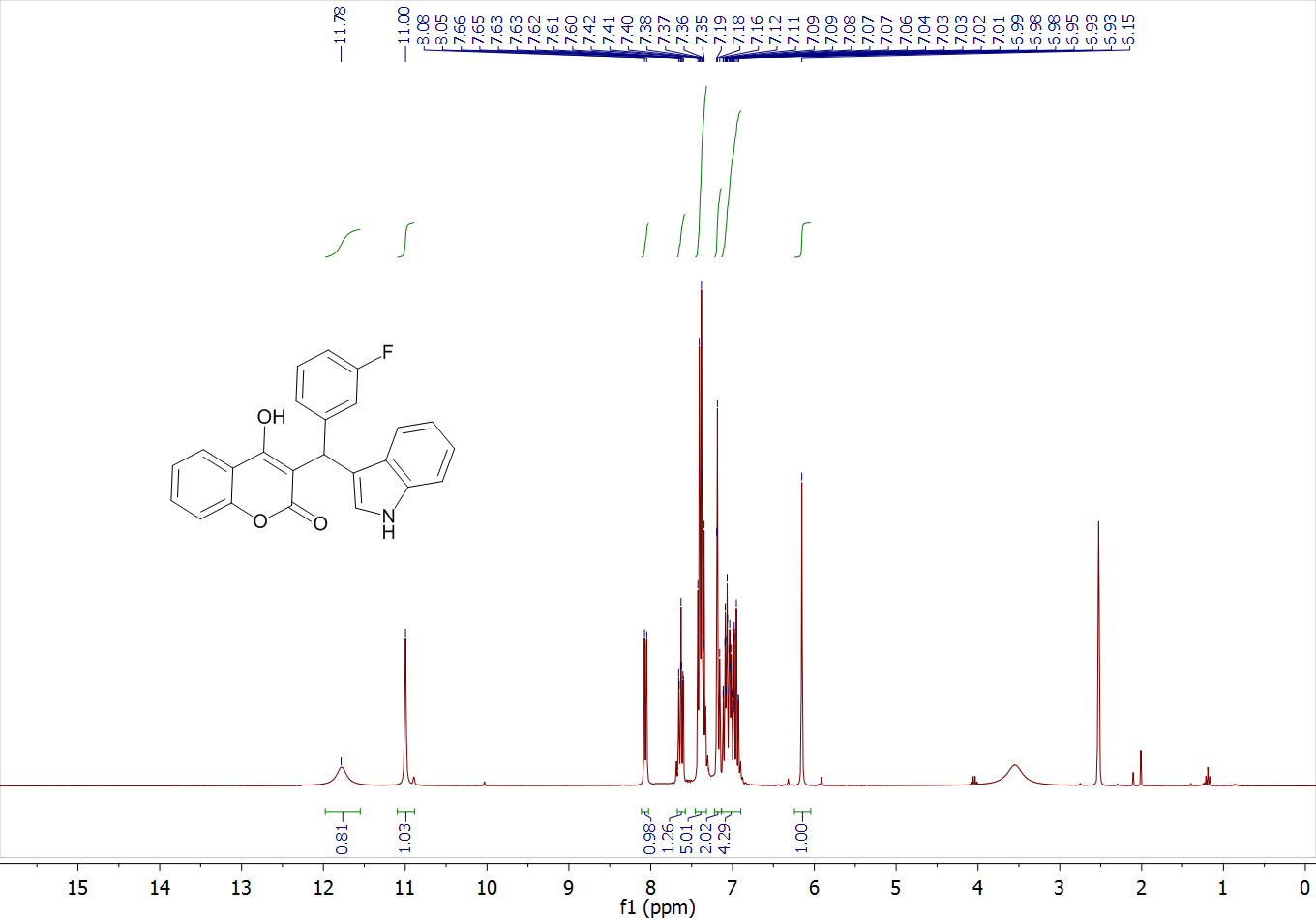
**

**
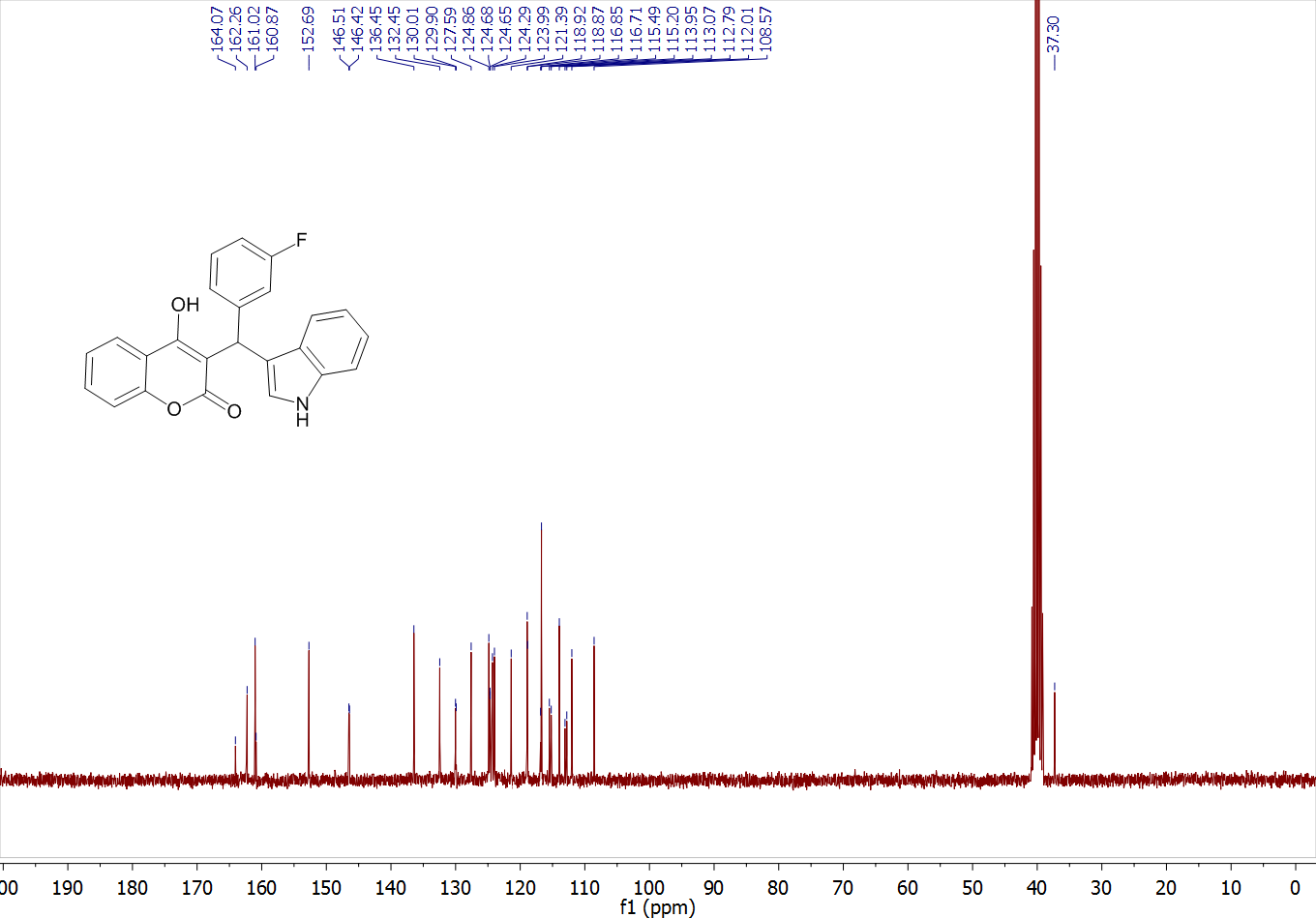
**

**3-((4-fluorophenyl)(1*H*-indol-3-yl)methyl)-4-hydroxy-2*H*-chromen-2-one (4h)**

**
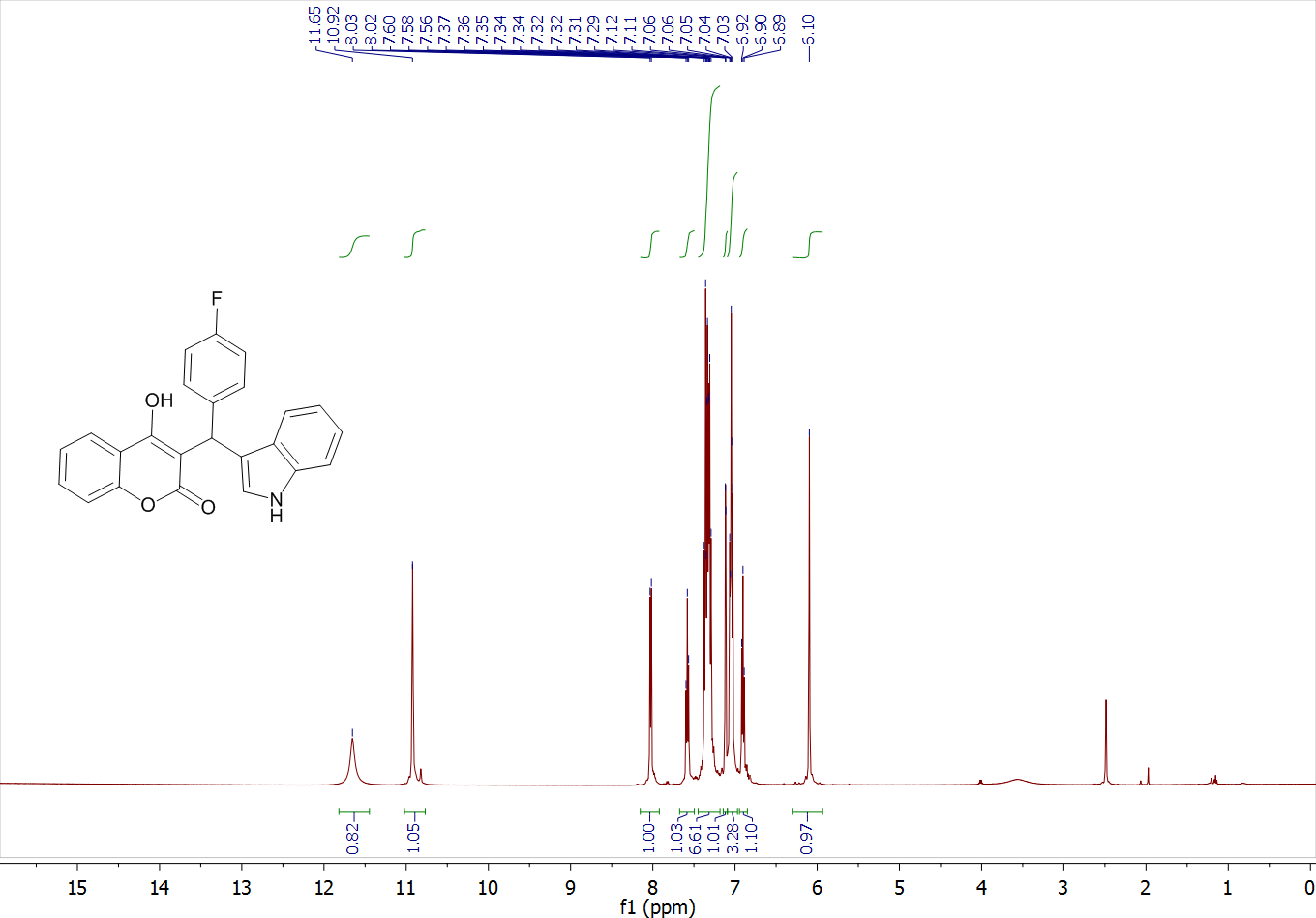
**

**
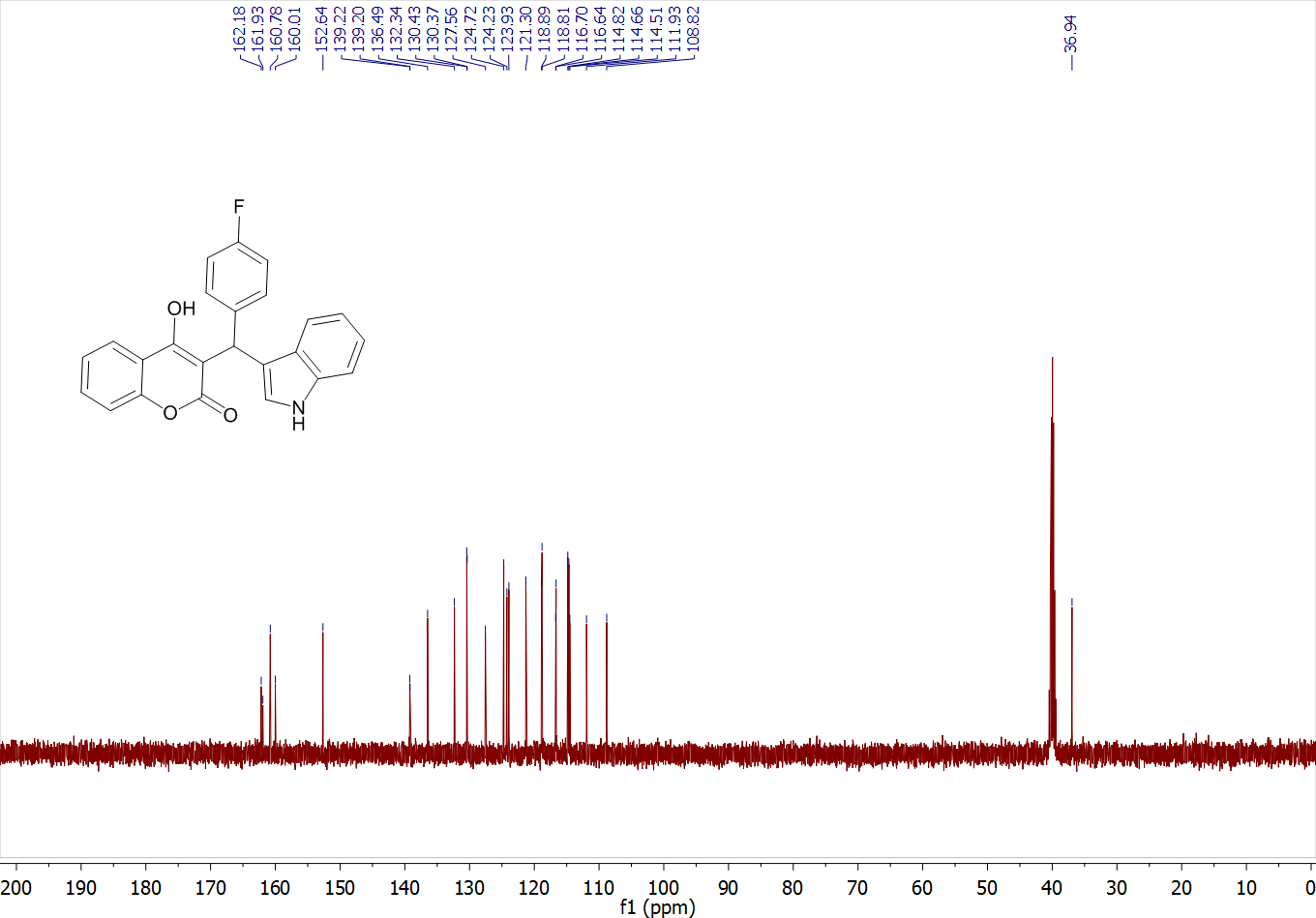
**

**3-((3-Chlorophenyl)(1*H*-indol-3-yl)methyl)-4-hydroxy-2*H*-chromen-2-one (4i)**

**
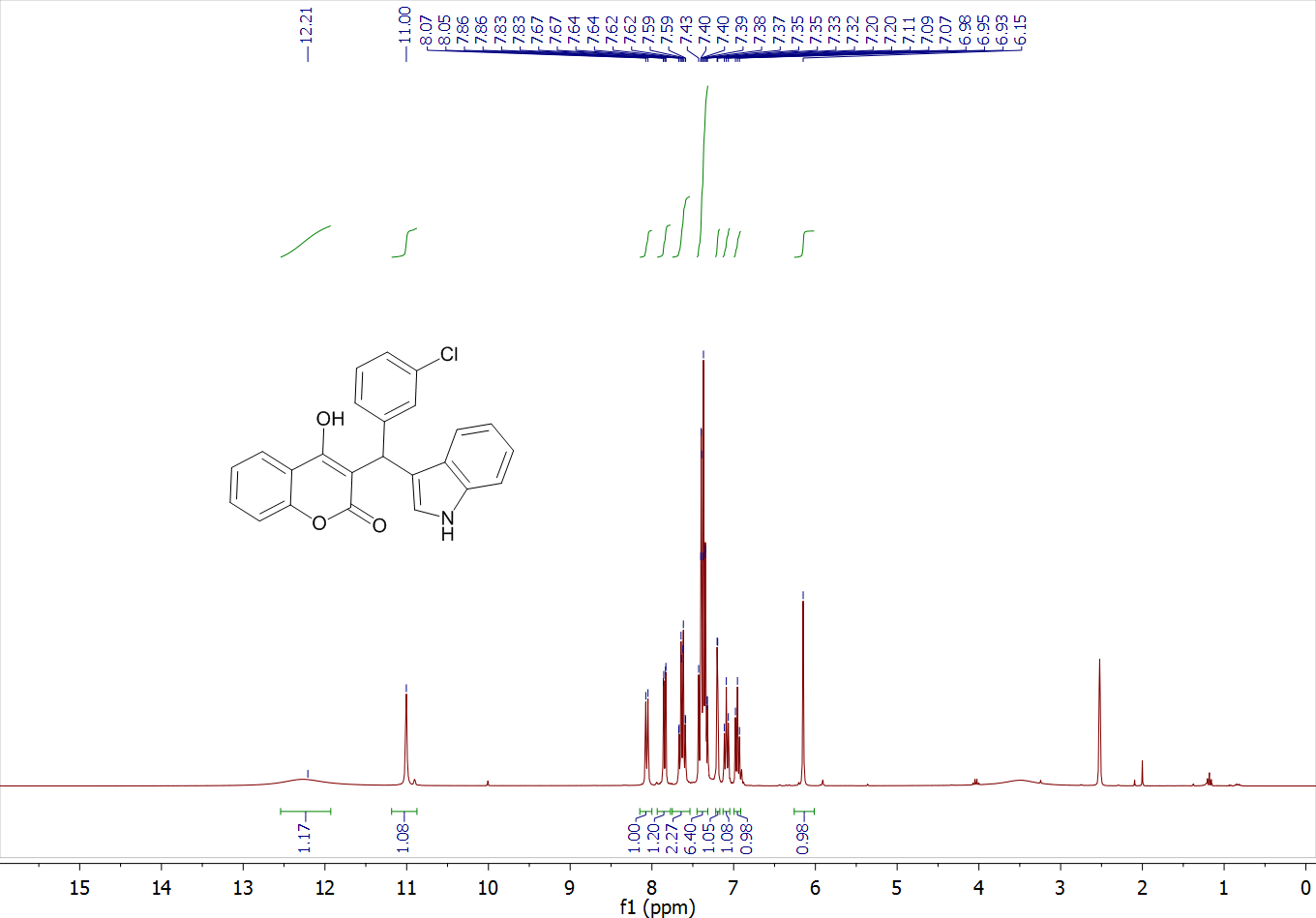
**

**
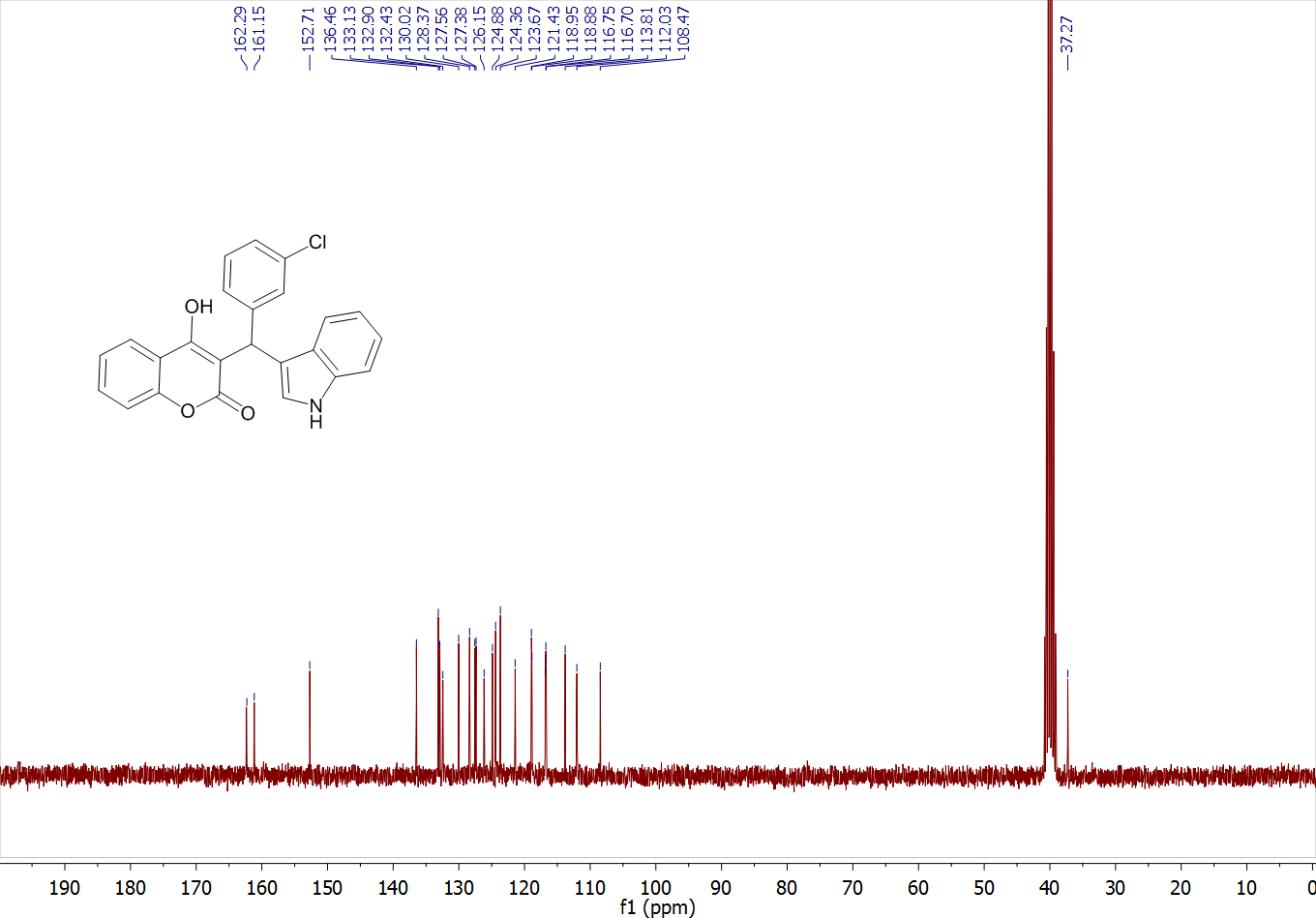
**

**3-((4-chlorophenyl)(1*H*-indol-3-yl)methyl)-4-hydroxy-2*H*-chromen-2-one (4j)**

**
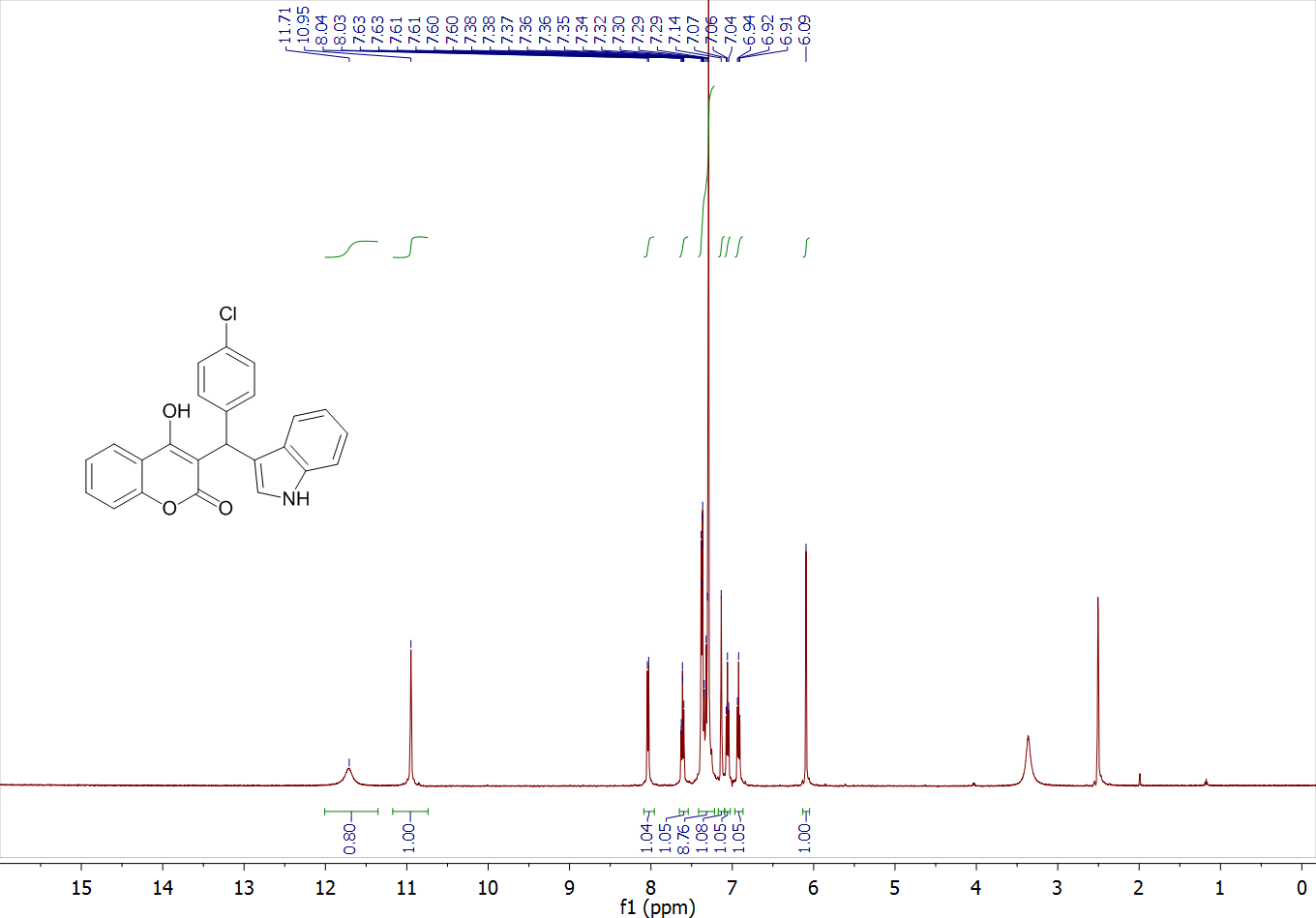
**

**
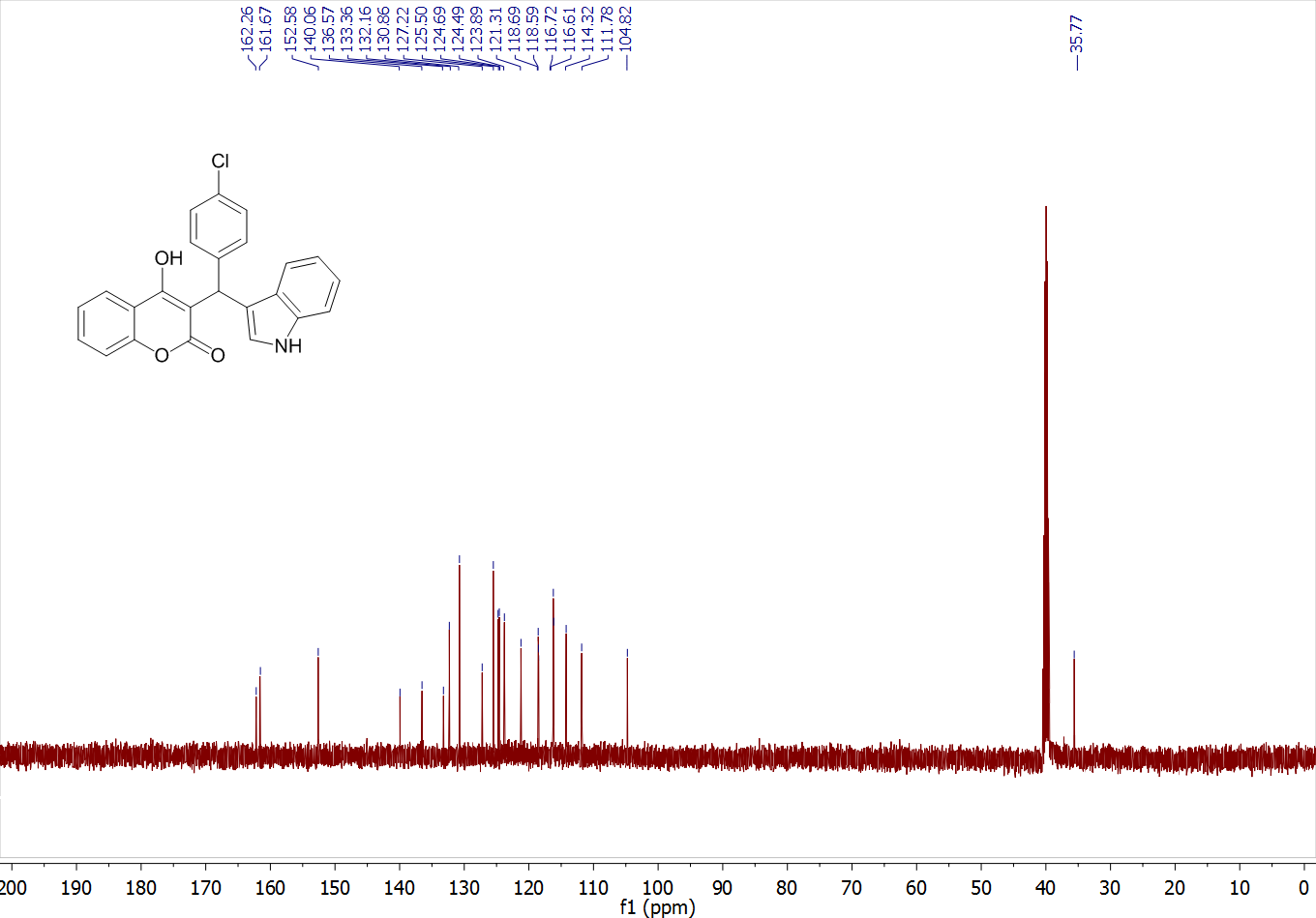
**

**3-((3-bromophenyl)(1*H*-indol-3-yl)methyl)-4-hydroxy-2*H*-chromen-2-one (4k)**

**
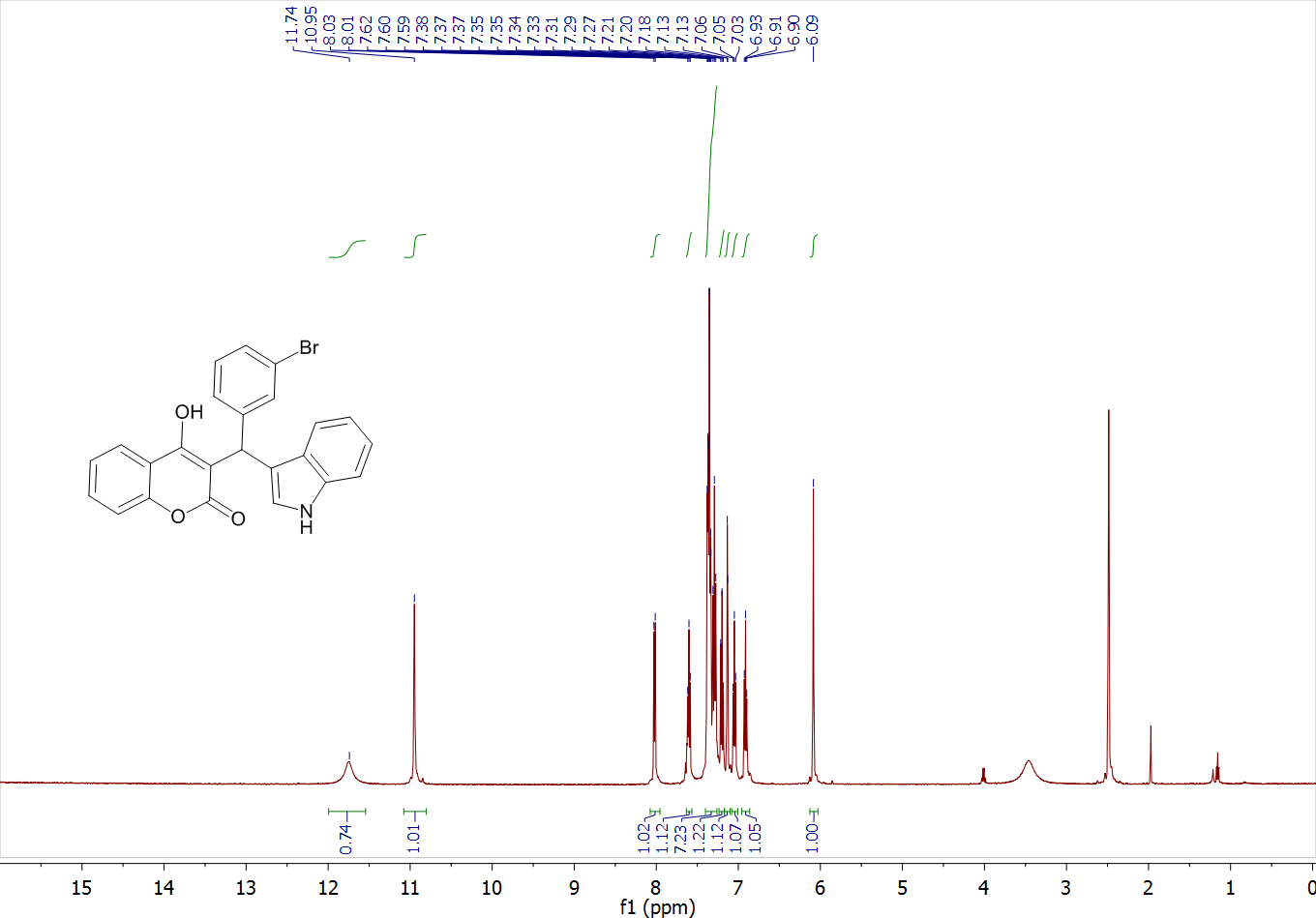
**

**
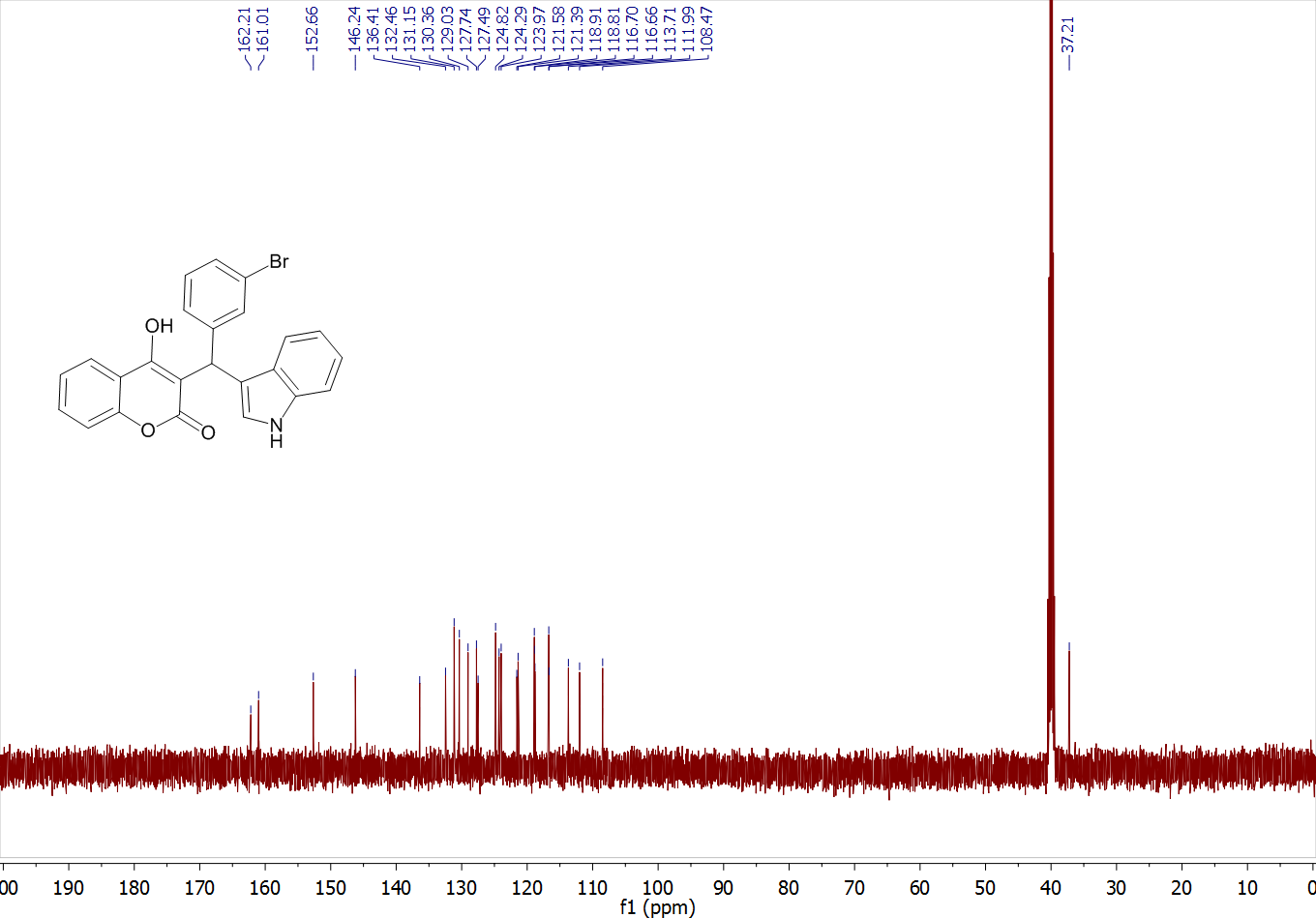
**

**3-((1*H*-indol-3-yl)(2-nitrophenyl)methyl)-4-hydroxy-2*H*-chromen-2-one (4l)**

**
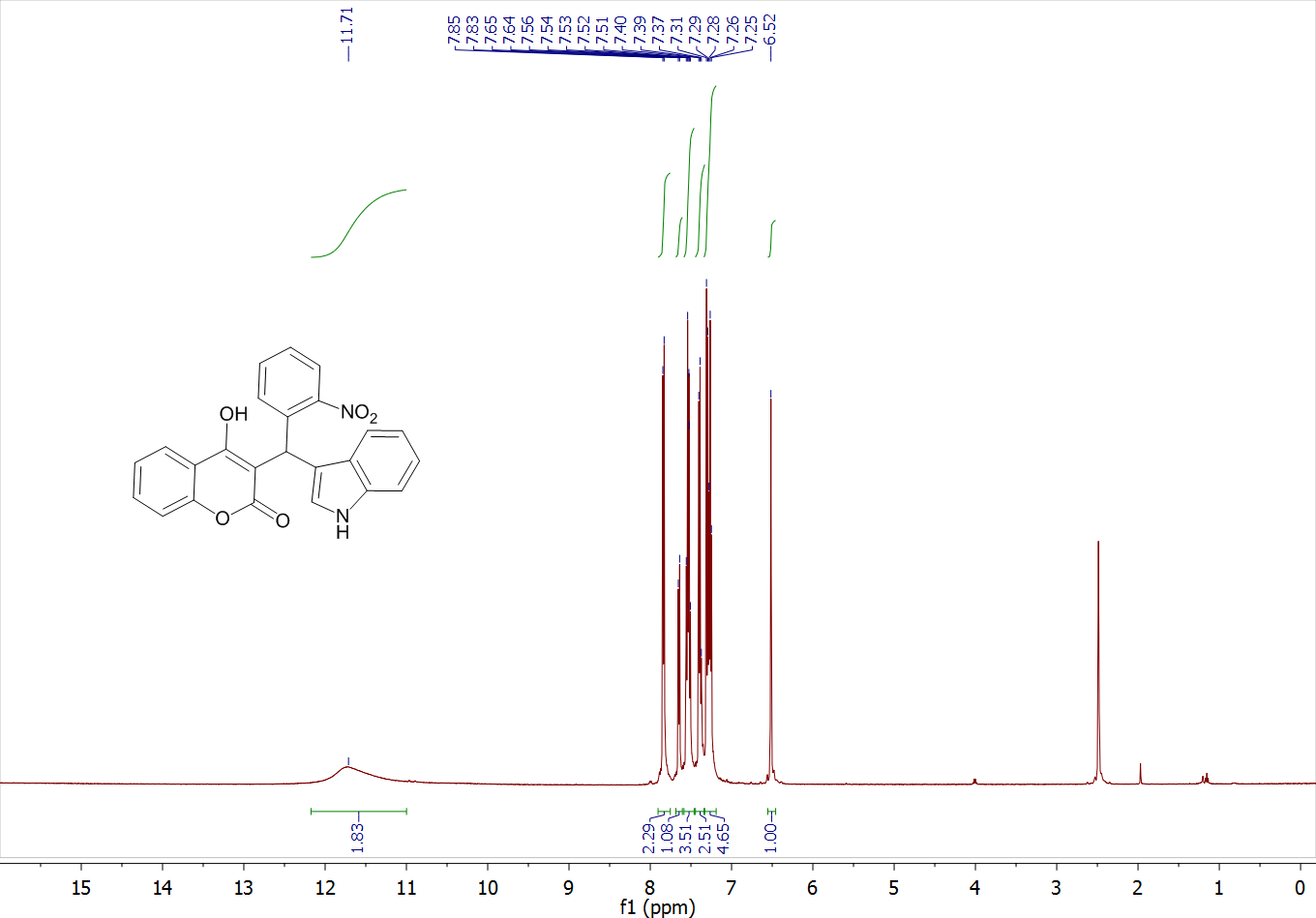
**

**
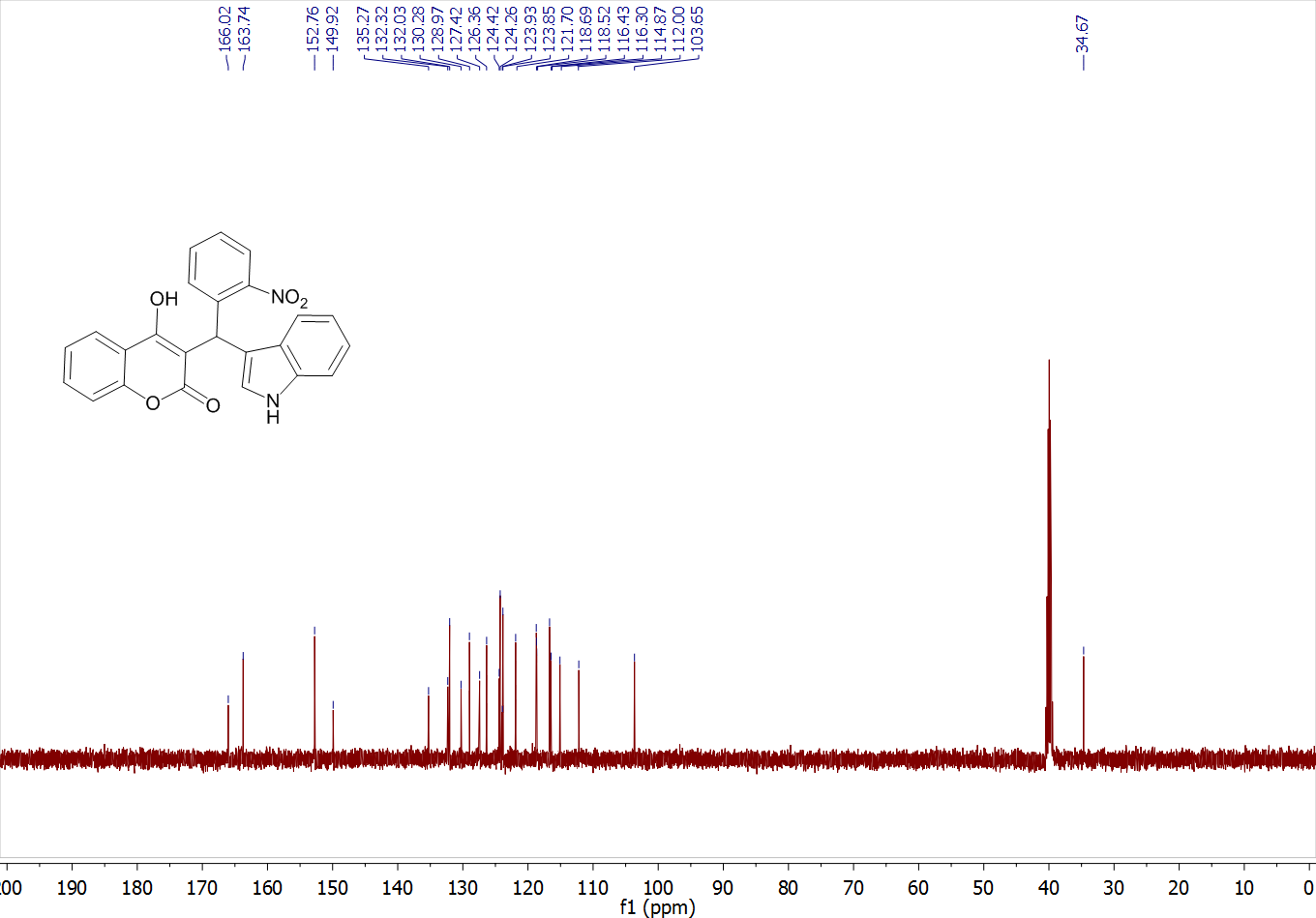
**

**3-((1*H*-indol-3-yl)(3-nitrophenyl)methyl)-4-hydroxy-2*H*-chromen-2-one (4m)**

**
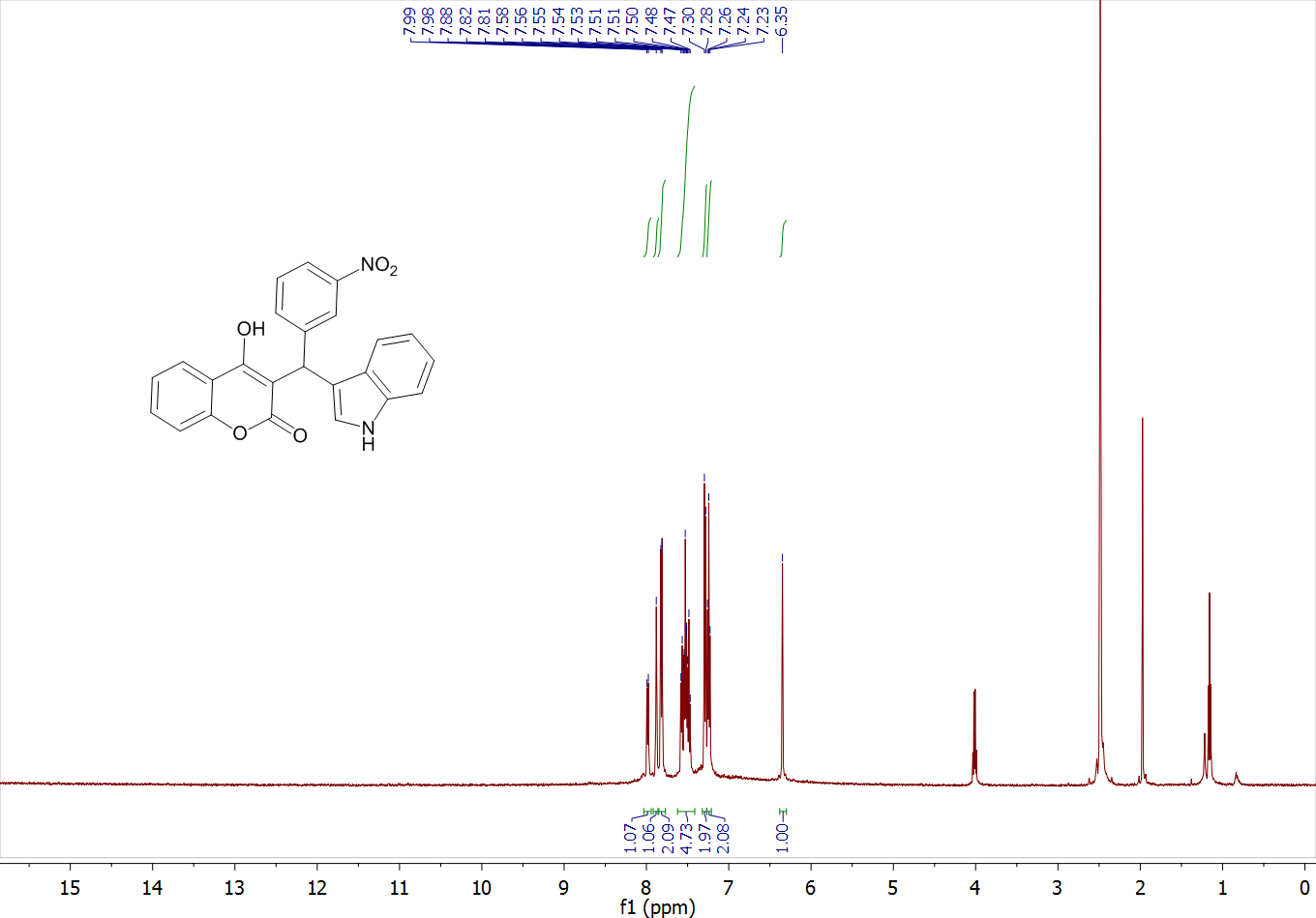
**

**
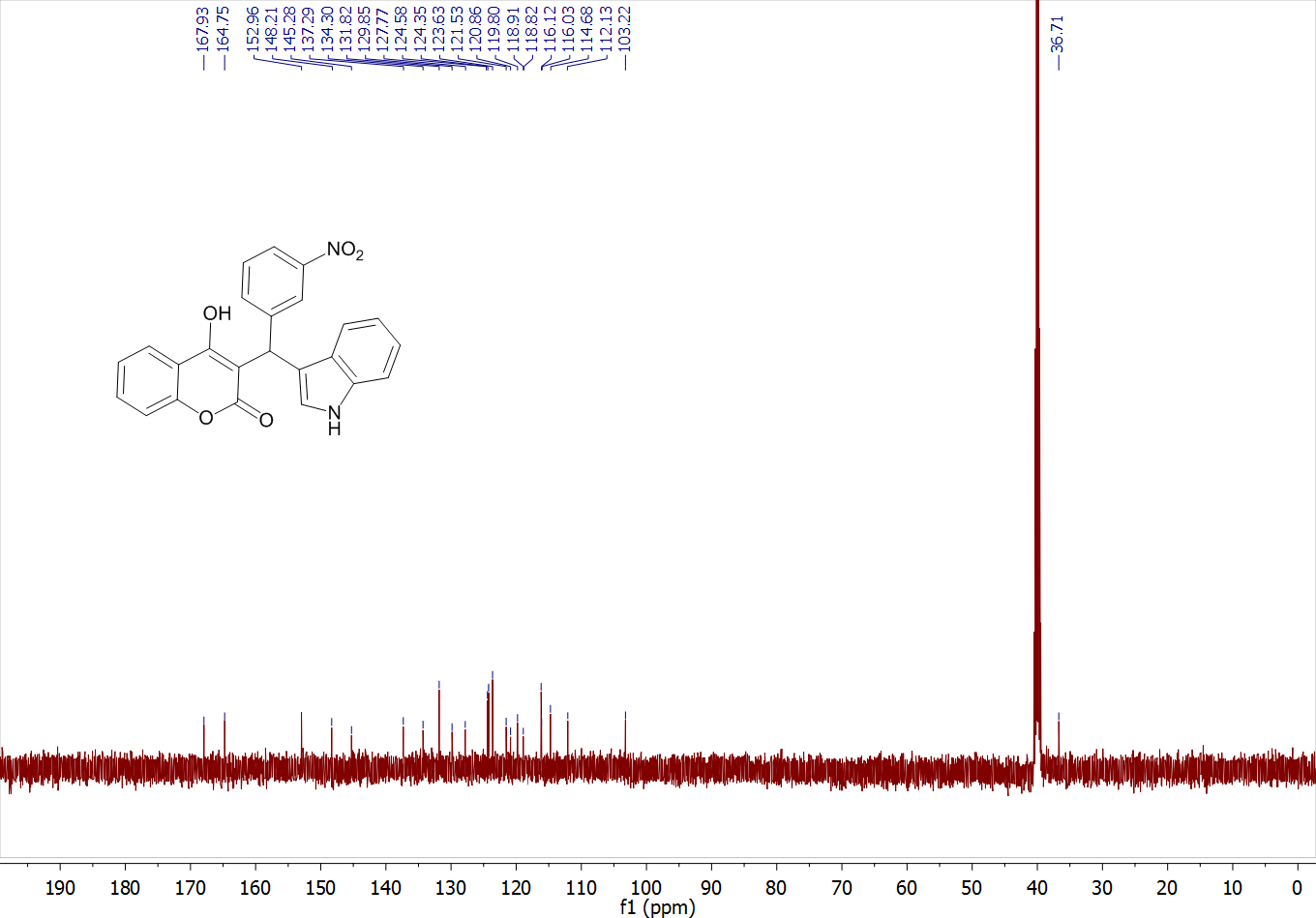
**

**
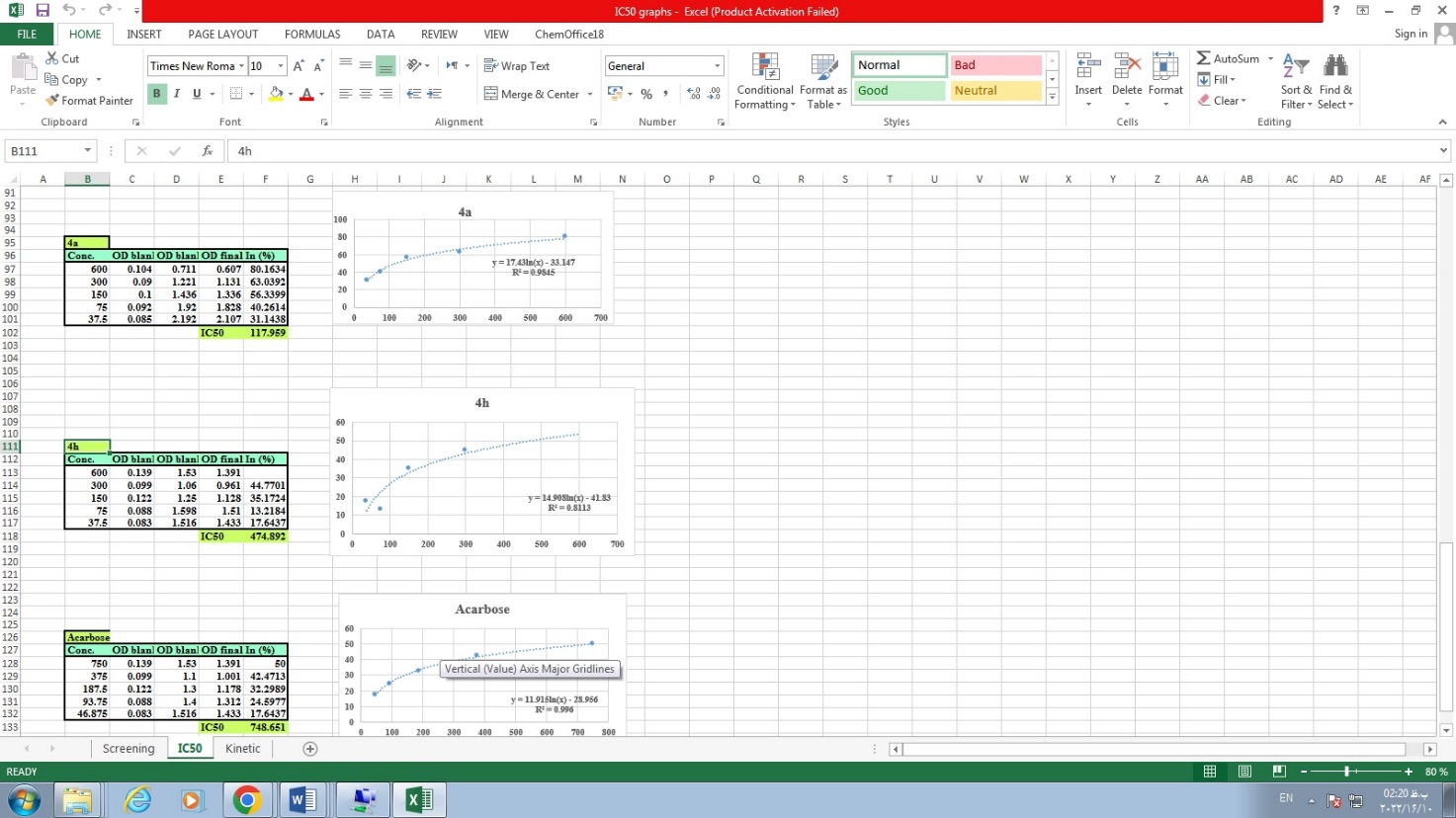
**

IC_50_ graphs for compounds **4a**, **4h**, and acarbose.

**
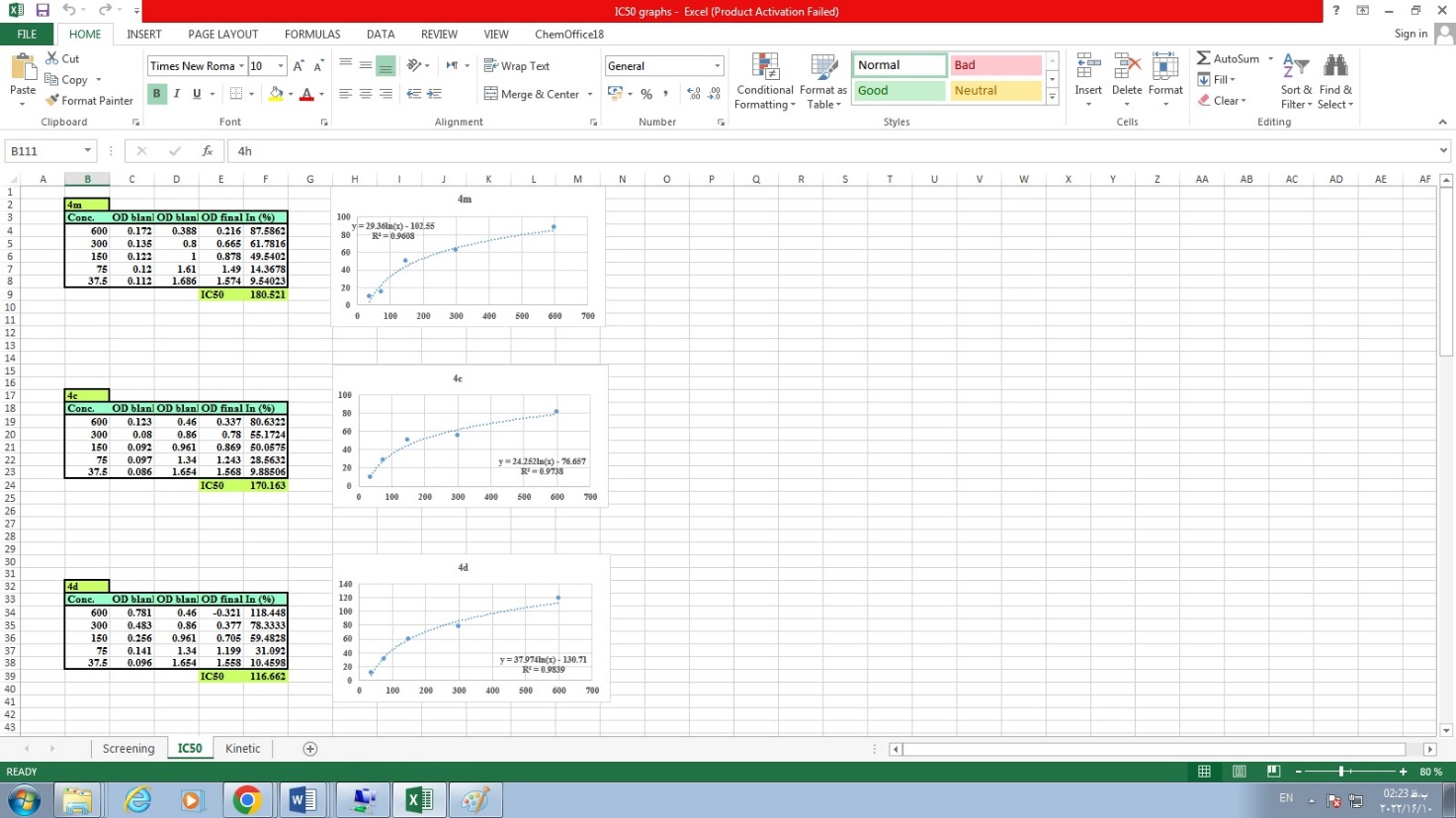
**

IC_50_ graphs for compounds **4m**, **4c**, and **4d**.

**
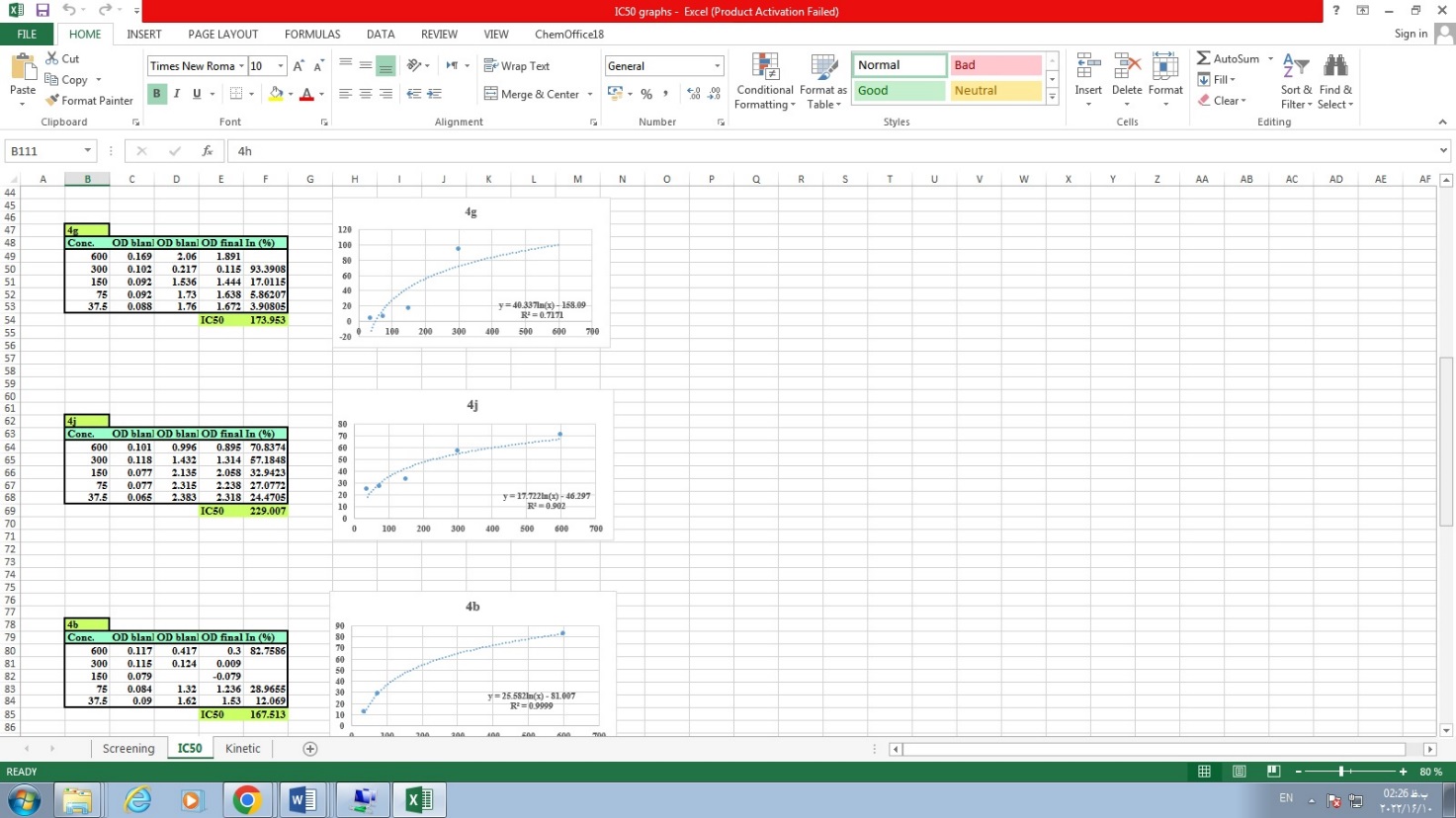
**

IC_50_ graphs for compounds **4g**, **4j**, and **4b**.

**
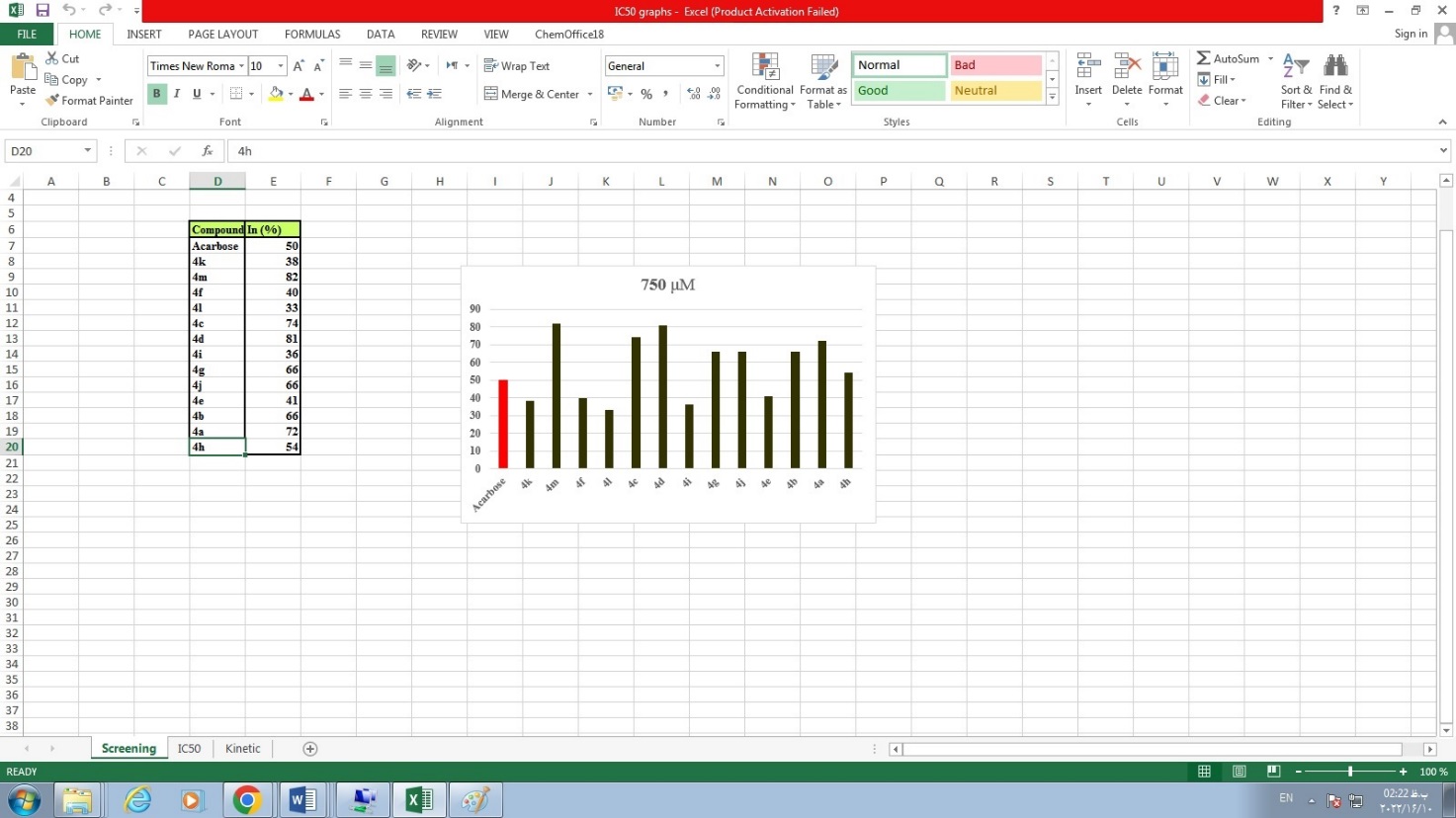
**

General screening of the title compounds

**
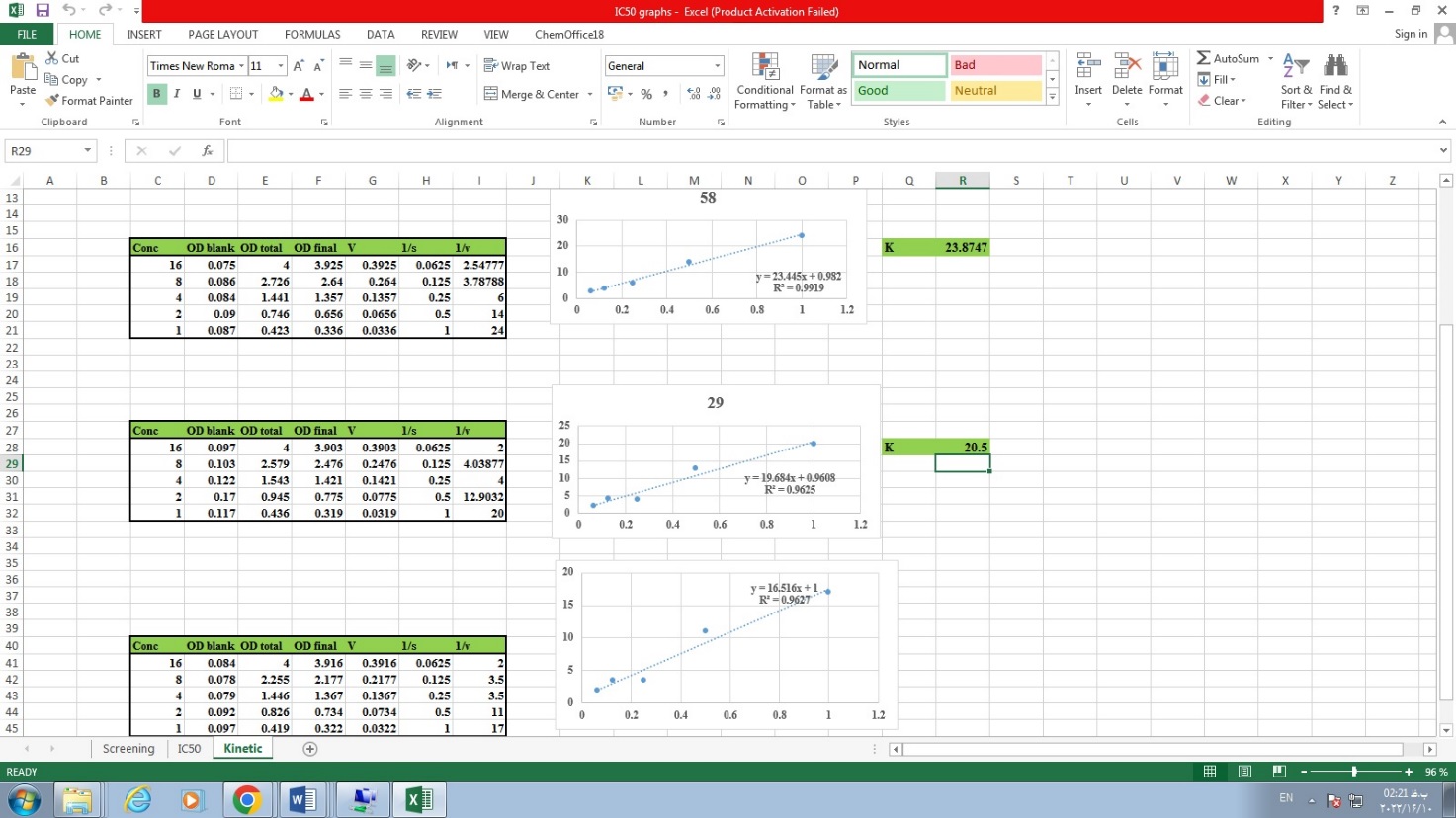
**

Kinetic study of compound **4d**
